# Whole-exome mutational landscape of neuroendocrine carcinomas of the gallbladder

**DOI:** 10.1038/s41392-020-00412-3

**Published:** 2021-02-10

**Authors:** Fatao Liu, Yongsheng Li, Dongjian Ying, Shimei Qiu, Yong He, Maolan Li, Yun Liu, Yijian Zhang, Qin Zhu, Yunping Hu, Liguo Liu, Guoqiang Li, Weihua Pan, Wei Jin, Jiasheng Mu, Yang Cao, Yingbin Liu

**Affiliations:** 1 Department of General Surgery, Xinhua Hospital Affiliated to Shanghai Jiao Tong University School of Medicine, Yangpu District, Shanghai, 200092 China; 2 Shanghai Key Laboratory of Biliary Tract Disease, Yangpu District, Shanghai, 200092 China; 3 State Key Laboratory of Oncogenes and Related Genes, Shanghai, China; 4 Shanghai Research Center of Biliary Tract Disease, Yangpu District, Shanghai, 200092 China; 5 Department of Biliary-Pancreatic Surgery, Renji Hospital Affiliated to Shanghai Jiao Tong University School of Medicine, Shanghai, China; 6 Department of Minimal Invasive Surgery, Ningbo Medical Center Lihuili Hospital, Ningbo, Zhejiang 315040 China; 7 Department of hepatopancreatobiliary surgery, Ganzhou hospital affiliated to Nanchang university, Jiangxi, 341000 China; 8 Department of Pediatric Surgery, Xinhua Hospital Affiliated to Shanghai Jiao Tong University School of Medicine, Yangpu District, Shanghai, 200092 China; 9 Information and Big Data Center, Xinhua Hospital Affiliated to Shanghai Jiao Tong University School of Medicine, Yangpu District, Shanghai, 200092 China; 10 Department of Surgery, Second Affiliated Hospital, School of Medicine, Zhejiang University, Hangzhou, Zhejiang, 310009 China; 11 Department of Gastric Surgery, Cancer Hospital of the University of Chinese Academy of Sciences, Hangzhou, Zhejiang 310022 China

**Keywords:** Gastrointestinal cancer, Cancer genomics, Cancer genomics

## Abstract

Neuroendocrine carcinoma (NEC) of the gallbladder (GB-NEC) is a rare but extremely malignant subtype of gallbladder cancer (GBC). The genetic and molecular signatures of GB-NEC are poorly understood; thus, molecular targeting is currently unavailable. In the present study, we applied whole-exome sequencing (WES) technology to detect gene mutations and predicted somatic single-nucleotide variants (SNVs) in 15 cases of GB-NEC and 22 cases of general GBC. In 15 GB-NECs, the C > T mutation was predominant among the 6 types of SNVs. *TP53* showed the highest mutation frequency (73%, 11/15). Compared with neuroendocrine carcinomas of other organs, significantly mutated genes (SMGs) in GB-NECs were more similar to those in pulmonary large-cell neuroendocrine carcinomas (LCNECs), with driver roles for *TP53* and *RB1*. In the COSMIC database of cancer-related genes, 211 genes were mutated. Strikingly, *RB1* (4/15, 27%) and *NAB2* (3/15, 20%) mutations were found specifically in GB-NECs; in contrast, mutations in 29 genes, including *ERBB2* and *ERBB3*, were identified exclusively in GBC. Mutations in *RB1* and *NAB2* were significantly related to downregulation of the RB1 and NAB2 proteins, respectively, according to immunohistochemical (IHC) data (*p* values = 0.0453 and 0.0303). Clinically actionable genes indicated 23 mutated genes, including *ALK*, *BRCA1*, and *BRCA2*. In addition, potential somatic SNVs predicted by ISOWN and SomVarIUS constituted 6 primary COSMIC mutation signatures (1, 3, 30, 6, 7, and 13) in GB-NEC. Genes carrying somatic SNVs were enriched mainly in oncogenic signaling pathways involving the Notch, WNT, Hippo, and RTK-RAS pathways. In summary, we have systematically identified the mutation landscape of GB-NEC, and these findings may provide mechanistic insights into the specific pathogenesis of this deadly disease.

## Introduction

Gallbladder cancer (GBC), a type of biliary tract cancer (BTC), accounts for 1.7% of all global cancer-related deaths.^1^ Neuroendocrine carcinoma (NEC) of the gallbladder (GB-NEC) is rare but more malignant than GBC, accounting for less than 1% of GBCs and is identified mostly in women.^2–4^ Given that the symptoms of GB-NEC are similar to those of other types of GBC, specific methods to distinguish it from other subtypes are currently lacking.^5^ As a result, pathologic studies using immunohistochemical (IHC) of biopsy tissue currently serve as the first-line tool to diagnose the disease in combination with routine imaging examinations, including ultrasound, CT and MRI, and general serum markers.^6^ To date, the common protein markers detected in tissue specimens involve chromogranin A (CgA), synaptophysin, and neuron-specific enolase,^7^ though their pathologic roles in the development of GB-NEC remain to be established. Since we lack sufficient knowledge on the pathologic mechanisms that govern the malignant transformation of GB-NEC, the only acceptable therapeutic modality for GB-NEC is the removal of the entire GB. In addition, in many cases, lymphadenectomy and liver partial lobectomy are used as complementary approaches to prevent reoccurrence.^8^ In the absence of the pathologic signature specific to GB-NEC, GB-NEC does not respond well to traditional radiotherapy and chemotherapy.^9,10^ Therefore, it is urgent to reveal the molecular signature that contributes to the pathologic progression of GB-NEC and help improve molecular targeting strategies and adjuvant therapy following surgery.

We previously unveiled the mutation landscape of GBC by using whole-exome sequencing (WES) technology, and we specifically found that the ErbB signaling pathway is the most extensively mutated pathway in GBC.^11^ In addition, ErbB pathway mutations are correlated with poor patient outcomes.^11^ Strikingly, activated *ERBB2/ERBB3* mutations upregulate the expression of the immune checkpoint marker PD-L1 to induce the immune evasion of GBC.^12^ PD-L1 blockade enhances the efficacy of anti-ERBB therapy in GBC cells carrying an *ERBB2/ERBB3* mutation. Our studies have provided a theoretical basis for ERBB2/ERBB3 targeted treatment and immunotherapy for GBC in the future. In addition, we previously reported a case of small-cell GB NEC (GB-SCNEC) and investigated genome-wide somatic mutations in primary and metastatic tumors.^13^ It is evident that the substantial characteristics of GB-NEC mutations in a large population will yield great promise for the diagnosis and therapy of GB-NEC.

In the present study, we performed WES to detect mutations in formalin-fixed paraffin-embedded (FFPE) samples from 15 cases of GB-NEC and compared these mutations with those found in 22 cases of GBC. We studied the characteristics of all the mutations, including distribution and frequency, and mutations in tumor-associated genes and somatic single-nucleotide variants (SNVs) in GB-NEC and GBC. We also investigated the SMGs and copy number variations (CNVs) in GB-NECs. We have revealed the mutation characteristics of GB-NEC distinct from GBC that may offer novel therapeutic target potential for the diagnosis, prognosis, and treatment of this deadly disease.

## Results

### Characteristics of mutations and SMGs

**Fig. 1:**
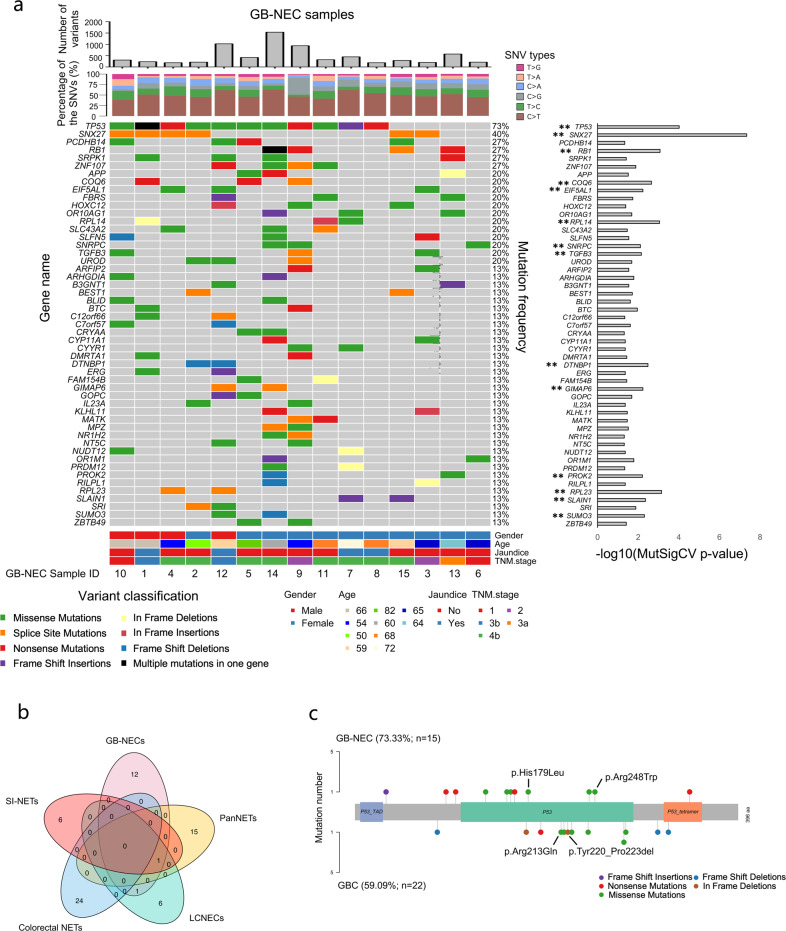
Characteristics of mutations in GB-NEC. **a** Name, mutation frequency, distribution in GB-NEC samples, and *p* value of significance of the SMGs with a MutSigCV *p* value < 0.05. Histograms of the total mutation number and fraction of the six types of SNVs in each GB-NEC sample are also shown. SMGs with *p* values < 0.01 are further highlighted and marked with two asterisks. **b** Overlap of SMGs detected in the five kinds of neuroendocrine cancers. **c** Different mutation sites of *TP53* in GB-NECs and GBCs. Known hotspot mutation sites in other tumors are labeled

To investigate mutations, we obtained average sequencing depths of 119.35× and 209.29× for GB-NEC and GBCs, respectively (Supplementary Fig. S1). Using the filter criteria described in the “Methods” section for functional mutations, we achieved a total of 6971 and 15,836 rare in the general population and nonsilent mutations in GB-NECs and GBCs, respectively. Detailed information on the mutations in GB-NECs is summarized in Supplementary Table S2. The average depth and alternative allele frequency of the mutations in GB-NECs were 117.36× and 31%, respectively. Supplementary Figure S2a shows the genome-wide distributions and basic information on the mutations, in which the mutations included 6338 SNVs and 633 insertions and deletions (INDELs) (Supplementary Fig. S2b). Missense mutations dominated in all of the mutation types (Supplementary Fig. S2c). The number of mutations in each sample ranged from 189 to 1539, with an average of 473.2 (Fig. 1a). In SNVs, the C > T mutation constituted the highest proportion (~50%) among the six types of base conversions, and transitions were significantly more common than transversions (Supplementary Fig. S2d, e). The fraction of the SNV in each sample was similar (Fig. 1a). Of note, *TP53* showed the highest mutation frequency of 73% (11/15) and presented in all male patients (4/4) and in 63.6% (7/11) of female patients (Fig. 1a, Supplementary Fig. S2f). Consistent with our previous finding that *HMCN1* was mutated in the primary and metastatic tumors in a case of GB-NEC,^13^ a mutation in *HMCN1* occurred in 33% (5/15) of the samples, suggestive of the reliability of current data analyses. We also investigated the known common variants in tumors, also known as hotspot mutations, according to the COSMIC database and by referring to the study of Chen et al.^14^ In GB-NEC samples, we found nine mutations that were known hotspot mutations in tumors, including two mutations in *TP53* (p.Arg248Trp and p.His179Leu) and two mutations in *CTNNB1* (p.Ser33Cys and p.Ser37Phe) (Supplementary Table S3).

By using a cutoff *p* value < 0.05, MutSigCV predicted a total of 67 potential SMGs in GB-NEC samples. The name, *p* value of significance and mutation frequency of the SMGs are summarized in Fig. 1a and Supplementary Table S4. These SMGs included *TP53*, *SNX27*, *RB1*, *COQ6*, *EIF5AL1*, *RPL14*, *SNRPC*, and *TGFB3*. The 14 SMGs with *p* values < 0.01 are further highlighted and marked with two asterisks in Fig. 1a. Next, we compared these SMGs with those of the other four kinds of neuroendocrine cancers, including pancreatic neuroendocrine tumors (PanNETs),^15^ LCNECs,^16^ colorectal neuroendocrine tumors (colorectal NETs),^17^ and small intestine NETs (SI-NETs).^18^ As shown in Fig. 1b and Supplementary Table S5, we found that the SMGs varied in these five kinds of NECs lacking a significant level of common genes. Only were *TP53* identified in GB-NECs, PanNETs, LCNECs, and SI-NETs and *RB1* in GB-NECs and LCNECs. The result revealed that the driver genes of NECs in different organs are quite different, and SMGs in GB-NECs are more similar to those in LCNECs, with potentially crucial roles for *TP53* and *RB1*. We also validated the mutations of SMGs by reviewing with Integrative Genomics Viewer software manually. The validation results of several SMGs with high mutation frequency are shown in Supplementary Fig. S3. It should be noted that *SNX27* mutations were located in a continuous T base sequence of the splice site, and its mutation frequency and role in GB-NEC need to be verified in a larger cohort.

Similar to GB-NEC, the mutation characteristics of 22 cases of GBC was also identified and analogous with our previous reports (Supplementary Fig. S4). The average number of mutations in the GBC samples (731.5) was higher than that in the GB-NEC samples (473.2). In the SNVs, C > A was secondary to C > T mutations in GBCs, while the second most frequent mutation in GB-NEC was T > C. In addition, in GBCs, the mutation frequency of *TP53* was ranked second, at 59% (Supplementary Fig. S5, Supplementary Table S6). Ten mutations were known hotspot mutations, including *PIK3CA* p.Arg88Gln and p.Glu545Lys, *KRAS* p.Gly12Asp and p.Gly13Asp and *TP53* p.Arg213Gln and p.Tyr220_Pro223del (Supplementary Table S3). The individually divergent mutation locus of *TP53* in GB-NECs and GBCs is displayed on the different functional domains in Fig. 1c.

We were further concerned about the enriched signaling pathways of the mutated genes. The total number of mutated genes detected in at least one GB-NEC sample was 5071, of which 3047 genes were also mutated in GBCs, but 2024 were mutated specifically in GB-NECs (Supplementary Tables S2 and 6, Supplementary Fig. S6a). The genes mutated specifically in GB-NECs were mostly enriched in pathways in cancer and the JAK-STAT pathway (Supplementary Fig. S6b, Supplementary Table S7). The mutated genes detected in both GB-NECs and GBCs were found to be mostly enriched in the PI3K-Akt signaling pathway (Supplementary Fig. S6c, Supplementary Table S8). The data revealed that gene mutations in GB-NECs involved in signaling pathways had similarities and differences with those in GBCs, implicating the unique features of mutated pathways present in GB-NECs.

### Distinct mutational statuses of known cancer-related genes

**Fig. 2:**
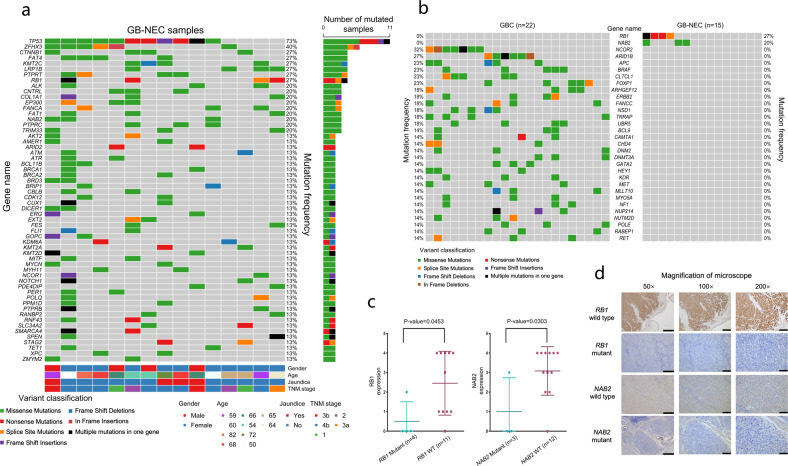
Distinct mutational statuses of known cancer-related genes. **a** Mutations in known cancer-related genes in the CGC database that were detected in more than one GB-NEC sample. **b** Mutations in genes mutated specifically in GB-NECs or GBCs. **c** Comparison of RB1/NAB2 protein expression between *RB1/NAB2* mutant and wild-type samples (*t* test). **d** IHC results at different magnifications from *RB1* wild-type and mutant samples; IHC results at different magnifications from *NAB2* wild-type and mutant samples. Scale bars in the figures with 50×, 100×, and 200× magnification indicate 500, 200, and 100 μm, respectively

To better understand mutations that may be relevant to carcinogenesis, we used the COSMIC Cancer Gene Census (CGC)^19^ database to analyze the mutation potential for promoting tumor development. In Tier 1 (*n* = 576) of the CGC database, a total of 58 cancer-related genes were mutated more than once in all 15 GB-NECs (Fig. 2a). Again, *TP53* showed the highest mutation frequency (73%), followed by 16 genes with a mutation frequency ≥20%: *ZFHX3* (40%), *CTNNB1* (27%), *FAT4* (27%), *KMT2C* (27%), *LRP1B* (27%), *PTPRT* (27%), *RB1* (27%), *ALK* (20%), *CNTRL* (20%), *COL1A1* (20%), *EP300* (20%), *FANCA* (20%), *FAT1* (20%), *NAB2* (20%), *PTPRC* (20%), and *TRIM33* (20%). Notably, the second most frequently mutated gene was *ZFHX3* (zinc finger homeobox 3), also known as *ATBF1* (AT motif binding factor 1), a transcription factor that induces neuronal differentiation and functions as a tumor suppressor in several types of tumors.

In parallel, we also analyzed the mutation status of Tier 1 cancer-related genes in 22 GBCs (Supplementary Fig. S7). Except for the fact that *TP53* was the frequently mutated gene (59%), identical to the earlier result in GBCs, we were particularly interested in the mutation frequencies of *ERBB2* and *ERBB3* (18 and 14%, respectively, analogous with our previous reports).^11,12^ In the comparison of mutated genes between GB-NECs and GBCs, *RB1* and *NAB2* were mutated frequently in GB-NECs (27%, 4/15, and 20%, 3/15, respectively) but not in GBCs (Fig. 2b). In contrast, 29 genes had a >10% mutation frequency in GBCs but were not mutated in GB-NECs. Such mutations included those in *NCOR2*, *ARID1B*, and others (Fig. 2b). Six of 15 (40%) GB-NEC samples had either an *RB1* or an *NAB2* mutation. Mutations in *RB1* included three stop-gain mutations (p.Gln689*, p.Ser634*, and p.Gln217*) and two splice-site mutations. Mutations in *NAB2* were all missense mutations, including p.Leu466Phe, p.Arg405His, and p.Ala4Thr (Supplementary Table S2). IHC analysis indicated that the expression of the RB1 protein was significantly lower in the 4 *RB1* mutated samples (*p* value = 0.0453) (Fig. 2c, d, Supplementary Fig. S8). Lower NAB2 expression was also observed in the three *NAB2* mutated samples (*p* value = 0.0303) (Fig. 2c, d, Supplementary Fig. S9). Figure 2d, Supplementary Fig. S8, and Supplementary Fig. S9 show the IHC results of RB1 and NAB2 in all of the GB-NEC samples. In concert with the distinct mutation patterns in GB-NEC and GBC, the mutation frequency of the *ERBB* family, including *EGFR* and *ERBB2/3/4*, was lower in GB-NECs (2/15, 13.3%) than in GBCs (6/22, 27.3%), although the *p* value (0.156) of the one-tailed chi-square test was not significant (Supplementary Fig. S10).

### Copy number variations (CNVs)

**Fig. 3:**
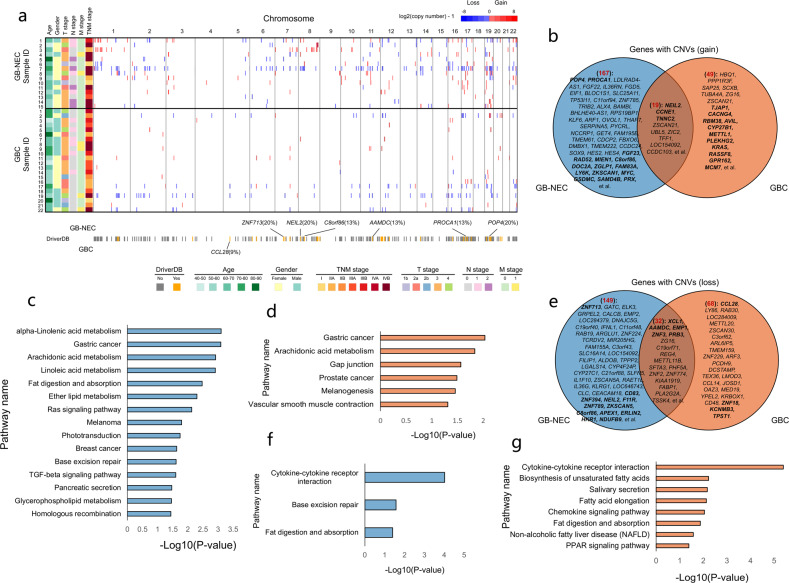
Genes with CNVs detected in GB-NECs. **a** Genome-wide distribution of all the CNVs in each sample of the 15 GB-NECs and 22 GBCs accompanied by the clinical information of the samples. The potential driver CNVs known in the DriverDB database were highlighted by color blocks with black borders in the heatmap. The CNVs that are absent in DriverDB were represented by color blocks without black borders. In addition, the genes with high-frequency potential driver CNVs in GB-NECs and GBCs were labeled. **b** Overlap of genes with a copy number gain detected in GB-NECs and GBCs. The name of genes with high-frequency CNVs was labeled in the Venn figures. Genes with potential driver CNVs were also labeled and highlighted in bold font. **c**, **d** Results of pathway enrichment analysis on the genes with a copy number gain in GB-NECs (**c**) and GBCs (**d**), respectively. **e** Overlap of genes with a copy number loss detected in GB-NECs and GBCs. **f**, **g** Results of pathway enrichment analysis on the genes with a copy number loss in GB-NECs (**f**) and GBCs (**g**), respectively

In GB-NECs, we identified a total of 186 genes with a copy number gain and 181 genes with a copy number loss. Among these genes with CNVs, 18 genes with a copy number gain and 17 genes with a copy number loss were identified as driver CNVs in other tumors recorded in the DriverDB (http://driverdb.tms.cmu.edu.tw/download) (Supplementary Table S9). The genome-wide distribution of these CNVs is shown in Fig. 3a, which indicated the variety of numbers and distributions of CNVs among the samples. We analyzed the distribution of the potential driver CNVs among the 15 GB-NEC samples and found that *POP4* (20%), *NEIL2* (20%), *ZNF713* (20%), *PROCA1* (13%), *C8orf86* (13%), and *AAMDC* (13%) had high-frequency CNVs (Fig. 3a). Moreover, in the known driver CNVs found in GB-NECs, the copy number of *MYC* was the highest (copy number 69.21) (Supplementary Table S9). In GBCs, a total of 68 genes with a copy number gain and 100 genes with a copy number loss were identified, among which 14 genes with a copy number gain and 9 genes with a copy number loss were identified as driver CNVs in the DriverDB (Fig. 3a, Supplementary Table S10). The CNVs of *CCL28* were observed to affect two GBC patients (Fig. 3a).

Next, we studied all the genes with CNVs in GB-NECs and compared them with those in GBCs. We found that in the 186 genes with a copy number gain detected in GB-NECs, 19 genes were shared with those in GBCs. In the 181 genes with a copy number loss detected in GB-NECs, 32 genes were shared with those in GBCs (Fig. 3b, e). The name of genes with high-frequency CNVs was labeled in the figures and genes with potential driver CNVs were highlighted by bold font. Functional enrichment analysis of genes with a copy number gain showed that several pathways including arachidonic acid metabolism etc. were shared by GB-NEC and GBC. There were also signaling pathways that were specifically enriched in GB-NEC, including Ras signaling pathway, base excision repair, TGF-beta signaling pathway, etc. (Fig. 3c, d, Supplementary Tables S11, and S12). Correspondingly, Fig. 3f, g shows the enriched signaling pathways of the genes with a copy number loss. Two signaling pathways, cytokine–cytokine receptor interaction and fat digestion and absorption were shared by GB-NEC and GBC, while base excision repair pathway occurred specifically in GB-NEC (Supplementary Tables S13 and S14). In addition, combined enrichment analysis of genes with copy number gain and loss in GB-NECs and GBCs are shown in Supplementary Fig. S11. The data revealed that CNVs in GB-NECs had similarities and differences with those in GBCs.

### Clinical relevance of genes and pathways with genomic alterations

**Fig. 4:**
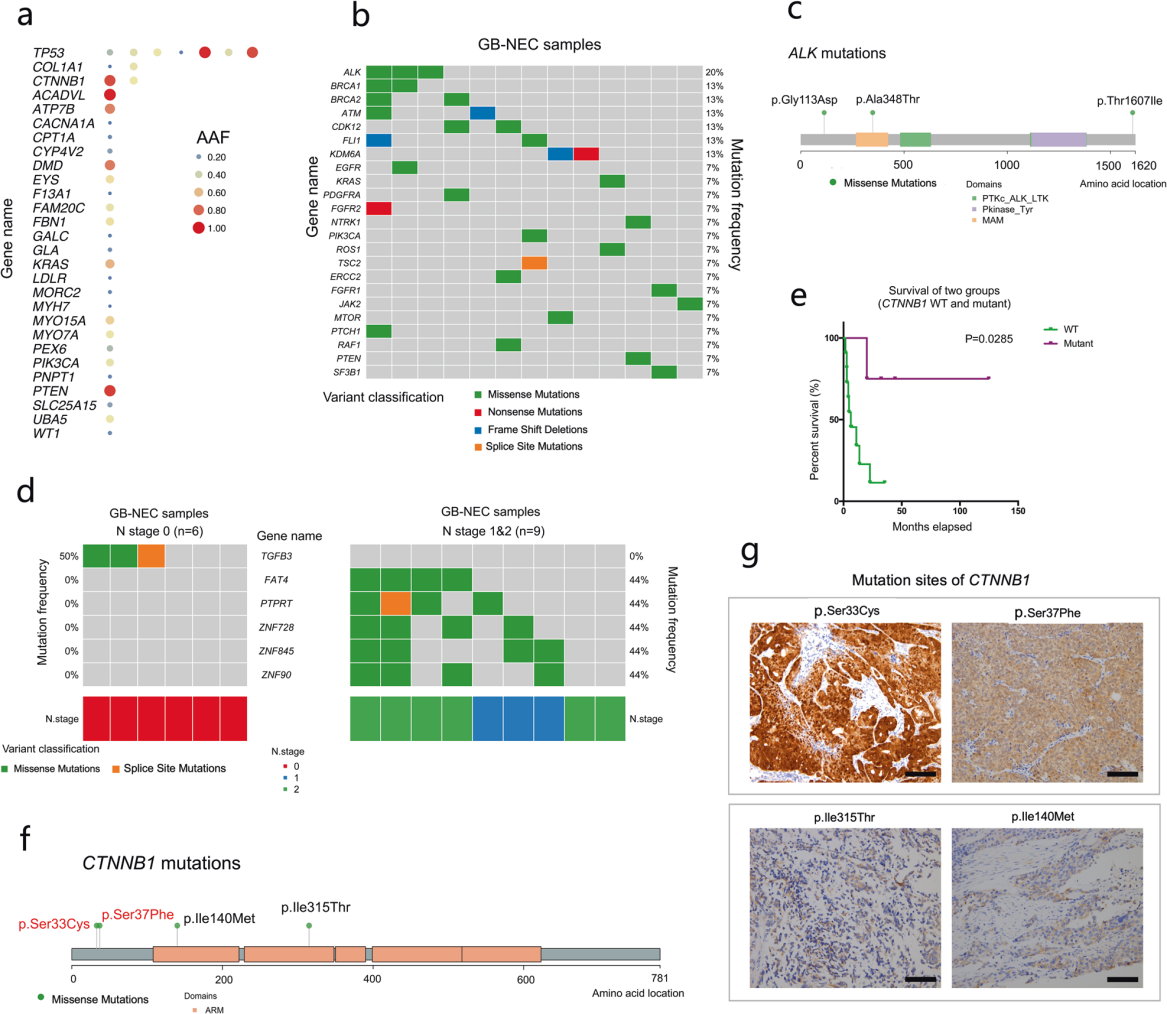
Clinical relevance of mutated genes in GB-NECs. **a** Genes with disease-related mutations detected in GB-NECs. **b** Detailed mutation information on genes with potential clinically actionable mutations recorded in the OncoKB database. **c** Detailed mutation sites of *ALK*. **d** Genes mutated specifically in stage N0 and N1/N2 GB-NECs. **e** Survival analysis of patients with *CTNNB1* mutations compared with those without *CTNNB1* mutations. **f** Detailed mutation sites of *CTNNB1*. **g** IHC results of β-catenin in the four GB-NEC samples with *CTNNB1* mutations. Scale bars in the figures indicate 100 μm

To evaluate the mutation potential for clinical relevance, we first focused on potentially functional and known disease-related variants in the ClinVar database (recorded as pathogenic or likely pathogenic). In 15 GB-NECs, we detected a total of 36 disease-related variants in 28 genes, in which *TP53* harbored 7 mutations, *COL1A1* and *CTNNB1* had 2 mutations, and the remaining genes possessed 1 mutation (Fig. 4a, Supplementary Table S15). In 22 GBCs, we found 71 genes with disease-related variants (Supplementary Fig. S12a and Supplementary Table S16), in which 10 genes overlapped with those in GB-NECs (Supplementary Fig. S12b).

In 55 actionable genes recorded in the OncoKB dataset^20^, 23 genes with potential drug responses were mutated in 86.67% (13/15) of patients with GB-NEC (Fig. 4b). The top seven genes, namely, *ALK*, *BRCA1*, *BRCA2*, *ATM*, *CDK12*, *FLI1*, and *KDM6A*, were mutated in more than one patient. The mutations *ALK* p.Gly113Asp, p.Ala348Thr and p.Thr1607Ile are displayed in Fig. 4c. We also further annotated these mutations with the OncoKB levels (levels 1–4 indicate a response to drugs, and levels R1/R2 indicate resistance to drugs) and reported cancer types and drugs recorded in the OncoKB dataset (Supplementary Table S17). Correspondingly, in GBC patients, 40 genes with potential drug responses were mutated in 95.45% (21/22) of tumors. The mutation frequencies of *BRAF*, *MTOR*, *ERBB2*, *PIK3CA*, and *ATM* were the highest (Supplementary Fig. S13, Supplementary Table S18).

We also analyzed the differences in mutated genes in GB-NEC tumors with different N stages. *TGFB3* was mutated only in the N0 group (*n* = 6); in contrast, *FAT4*, *PTPRT*, *ZNF728*, *ZNF845*, and *ZNF90* were mutated specifically in the N1/N2 group (*n* = 9) (Fig. 4d). We further studied the relationship between these N-stage-related genes and T/M stages in GB-NEC. We found that *TGFB3* mutations occurred only in stage T1/T2 GB-NECs (Supplementary Fig. S14). In 22 GBCs, genes mutated specifically in the N0 group (*n* = 16) included *RP1L1*, *LOXHD1*, *STAG2*, and *TCHH*, and those in the N1 group (*n* = 6) included *ANKRD28*, *CACNA2D3*, *EPB41L2*, *GNA14*, and others (Supplementary Fig. S15).

Survival analysis was performed on the genes with a mutation frequency ≥ 26.67% (4/15) in GB-NEC (Supplementary Table S2). For each gene, overall survival (OS) was compared between patients in whose tumors harbored the wild-type (WT) gene and those in whose tumors harbored the mutant gene. A mutation in *CTNNB1* (*n* = 4) was found to be related to prolonged OS (median OS: 38.25 vs. 6.33 months; *p* value = 0.0285) (Fig. 4e). Detailed mutation sites in *CTNNB1* are shown in Fig. 4f. By using IHC, we detected the subcellular distribution of β-catenin in all of the GB-NEC samples. We found that two of the *CTNNB1* mutated samples (with *CTNNB1* p.Ser33Cys and p.Ser37Phe) showed nuclear expression of β-catenin (Fig. 4g). The other two *CTNNB1* mutated samples (with *CTNNB1* p.Ile315Thr and p.Ile140Met) showed no nuclear expression of β-catenin (Fig. 4g). In addition, all other 11 GB-NEC samples without *CTNNB1* mutations showed no nuclear expression of β-catenin (data not shown). We also re-examined the potential clinical relevance of all 58 genes presented in Fig. 2a. In addition to the positive relationship between *CTNNB1* mutation and the OS of patients mentioned earlier, we found that mutations in *MYH11*, *KDM6A*, and *XPC* occurred in the tumors of patients who experienced extremely poor OS (Supplementary Fig. S16). In addition, among the genes with a high frequency of CNVs, CNVs in *AAMDC* (*n* = 2) were related to the poor overall survival (median OS: 3.48 vs. 20.07 months; *p* value = 0.0258) of GB-NEC patients (Supplementary Fig. S17).

Next, we analyzed the clinical relevance of the enriched signaling pathways of genes mutated specifically in GB-NECs, as shown in Supplementary Fig. S6b. A total of seven pathways, including the prolactin signaling pathway, osteoclast differentiation, the JAK-STAT signaling pathway, the Hippo signaling pathway, signaling pathways regulating the pluripotency of stem cells, glycosaminoglycan degradation and influenza A, were investigated, and the combined mutation frequencies of mutated genes in these pathways were 73.3% (11/15), 73.3% (11/15), 86.7% (13/15), 80% (12/15), 73.3% (11/15), 33.3% (5/15), and 80% (12/15), respectively, in GB-NEC patients. However, the survival analysis indicated no significant associations between mutations in these pathways and the prognosis of GB-NEC patients (Supplementary Fig. S18).

### Potential somatic SNVs

**Fig. 5:**
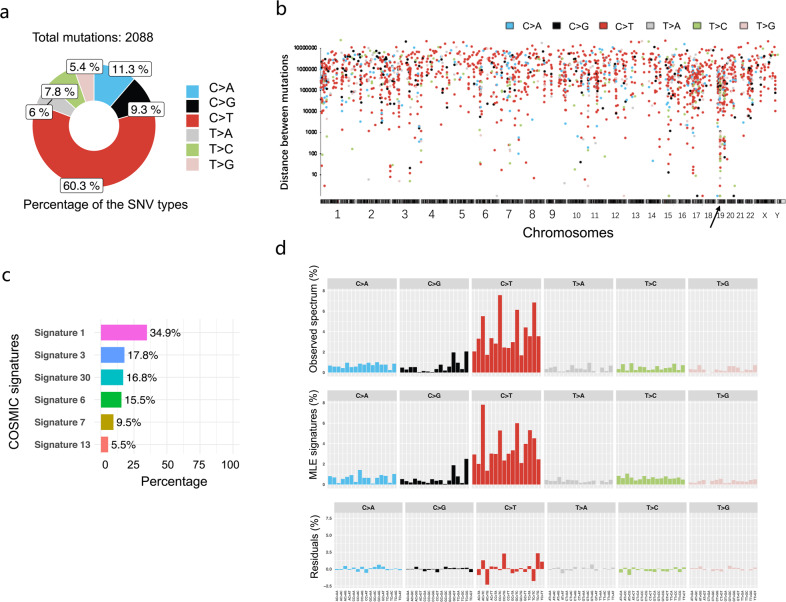
Characteristics of potential somatic SNVs. **a** Proportions of six SNV types in potential somatic SNVs. **b** Interdistance of the six types of SNVs. **c** Known COSMIC mutation signatures discovered in the potential somatic SNVs. **d** Comparison between the observed distribution of the somatic SNVs across the 96 possible mutation types and the summation of the distributions of the decomposed signatures

To determine somatic point mutations, we employed ISOWN and SomVarIUS software. A total of 2088 potential somatic SNVs from the rare mutations were predicted (Supplementary Table S19). Similar to earlier studies, in six types of SNVs, the C > T mutation dominated, accounting for 60.3% (Fig. 5a). We then analyzed the genome-wide distribution of all 2088 SNVs to define hypermutated regions (Fig. 5b). One “Kataegis”, the hypermutation region, was found in chromosome 19, and *ZNF43* was found to be in this “Kataegis” (Supplementary Table S19).

We then investigated the mutation signatures of the potential somatic SNVs. Decomposition analysis was performed using the 30 COSMIC signatures combined with ranking analysis using Bayesian information criterion (BIC). The mutation signatures varied in 15 GB-NEC samples (Supplementary Fig. S19). The decomposed signatures of all 2088 potential somatic SNVs from all samples revealed signatures 1, 3, 30, 6, 7, and 13 (Fig. 5c). Signature 1, the most common signature in multiple types of tumors that is related to the spontaneous deamination of 5-methylcytosine, contributed to the largest component (34.9%). A comparison of the distribution between the observed and decomposed signatures of 2088 SNVs across the 96 possible mutation types showed a cosine similarity of 0.949 and a BIC of 16887.720 (Fig. 5d), confirming the predominant somatic SNV of C > T.

**Fig. 6:**
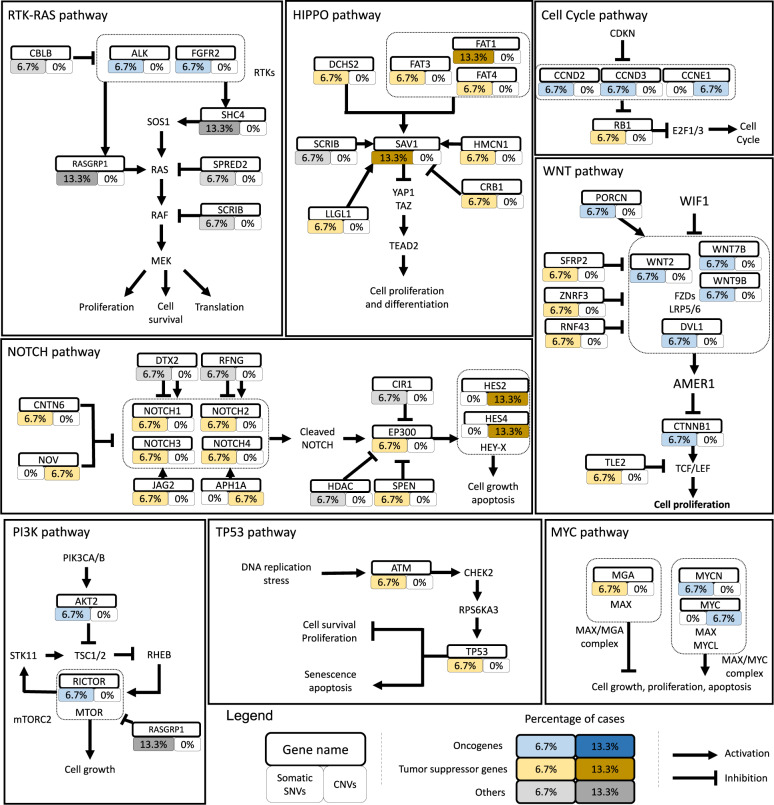
Potential somatically altered oncogenic signaling pathways in GB-NECs. According to the study of Francisco Sanchez-Vega et al.^21^, the frequencies of somatic SNVs and CNVs of important genes involved in the oncogenic signaling pathways are labeled to the regulatory network of proteins they encode

Finally, to explore the signature of SNVs related to oncogenic signaling pathways in TCGA cohorts, important members of ten key oncogenic pathways were analyzed according to the study of Francisco Sanchez-Vega et al.^21^ Genes carrying somatic SNVs were labeled on the signaling pathways based on the regulatory relationship between the encoded proteins as described by Francisco Sanchez-Vega et al.^21^ (Fig. 6). Most genes with potential somatic SNVs (12 genes) were enriched in the Notch pathway (Supplementary Fig. S20, Fig. 6). The other three pathways, the WNT, Hippo, and RTK–RAS pathways, carried 10, 9, and 7 genes with somatic SNVs, respectively (Fig. 6). To comprehensively understand the distribution of potential somatic SNVs or CNVs in the important genes of these pathways, we also included genes with CNVs in Fig. 6. Collectively, these data suggest that the primary form of somatic SNVs with C > T occurs in a number of oncogenic pathways, such as the Notch, WNT, Hippo, and RTK–RAS pathways.

## Discussion

GB-NEC is a rare but highly malignant subtype of GBC. Its pathologic progression may be associated with neuroendocrine dysfunction but remains to be fully characterized. To date, molecular targeted therapy is still unavailable. Eric Raymond et al.^22^ studied PanNETs and found that sunitinib, a multitargeted receptor tyrosine kinase (RTK) inhibitor, can improve patient progression-free survival, overall survival, and drug responses. Therefore, it is undoubtedly of therapeutic value to fully reveal the molecular signatures of GB-NEC. In the present study, we systematically studied mutations in GB-NECs for the first time. In comparison with GBC, we described the basic characteristics of mutations and the most frequently mutated genes and pathways that likely mediate the progression of GB-NEC. We investigated the SMGs and CNVs in GB-NECs. We also predicted potential somatic SNVs and analyzed somatic mutation signatures and oncogenic signaling pathways in GB-NECs. These findings have advanced our current knowledge and provided global insights into the genetic signature of GB-NEC, assisting in clinical diagnosis, prognosis, and potential therapy.

We successfully recruited 15 cases of GB-NEC from multiple clinical practice sites and performed an overall analysis of the functional mutations with population frequency and detailed impact annotations of the mutations. The analyses of genome-wide mutations made it possible to globally investigate the characteristics of the mutations and the mutation frequency of genes. In addition, although a lack of normal GB tissues made it difficult to distinguish somatic mutations from germline mutations, ISOWN and SomVarIUS were used to predict somatic SNVs, rendering it possible to analyze somatic mutation signatures and reveal mutated oncogenic pathways. We also screened the mutated genes that correlated with the clinical outcomes of patients, as case reports of GB-NECs associated with the clinicopathologic status were documented previously.^23,24^

It was not surprising to see *TP53* as the most frequently mutated gene in GB-NEC (11/15, 73%), indicating the reliability of our current methodology engaged in the mutagenesis study. *HMCN1* was frequently mutated (5/15, 33%), consistent with our previous study that identified *HMCN1* mutations in primary and metastatic GB-NECs, implying a tumor suppressive function for this gene.^13^ *HMCN1* was originally determined to be associated with age-related mechanical generation.^25^ In line with our findings, Zhao et al. demonstrated that *HMCN1* was frequently mutated in Chinese patients with prostate adenocarcinoma (PRAD).^26^ *HMCN1* is believed to be one of the key mutated genes connected to the recurrence of PRAD.^26^ Therefore, *HMCN1* may serve as a tumor suppressor in GB-NEC.

In COSMIC, we found a high mutation frequency of *ZFHX3* (40%). *ZFHX3* is a tumor suppressor transcription factor,^27^ and its mutations are associated with the outcome of endometrial cancer patients. Walker et al.^28^ found that patients with high-grade endometrial tumors expressed *ZFHX3* mutations and tended to have frequent lymphovascular space invasion. Consistent with these data, Hu et al.^29^ found that *ZFHX3* inhibited prostate cancer cell proliferation by downregulating MYC. Although the mutations in *ZFHX3* need to be functionally characterized with cancer development, targeting *ZFHX3* mutations may hold therapeutic promise in GB-NEC treatment.

It is of great attention that *RB1* and *NAB2* had high mutation frequencies (27% and 20%, respectively) in GB-NEC but were not mutated in GBCs. These interesting data support our previous study that defined low somatic mutation frequencies of *RB1* and *NAB2* (3.8% and 1.3%, respectively) in 157 GBC samples.^12^ Our results also indicate that mutations in *RB1/NAB2* are associated with the downregulation of the RB1/NAB2 protein in cancer tissue. Therefore, low expression levels of mutated RB1 and NAB2 are specific biomarkers for GB-NEC. Moreover, *RB1* was predicted to be an SMG in our study. Consistent with our findings, the loss of RB1 has been found in a number of other NECs. For example, prostate small-cell NEC and LCNEC harbor frequent *RB1* mutations (47%), comparable with those in *TP53* (85%).^30,31^ These studies revealed the predictive value of *RB1* mutations in the chemotherapy resistance of these cancers. Hence, it is worthwhile to further investigate the mechanistic roles of *RB1* and *NAB2* in the malignant transformation of GB-NEC.

Several genes with high-frequency and important CNVs, including *POP4*, *CCNE1*, and *MYC*, have been observed in GB-NECs. *POP4* (POP4 homolog, ribonuclease P/MRP subunit), with the highest amplification frequency in GB-NECs (20%), is a protein-coding gene involved in the processing of precursor RNAs. Frequent amplification of *POP4* on 19q12 has also been observed in grade III breast cancers^32^ and periampullary adenocarcinomas.^33^ Silencing *POP4* reduces cell viability in grade III breast cancer cells harboring its amplification.^32^ *CCNE1* amplification was observed in both GB-NECs and GBCs in our study. Interestingly, *CCNE1* is also located at 19q12 and is frequently coamplified with *POP4*.^32,33^ Indeed, in one GB-NEC sample (GBNEC_4), *CCNE1* and *POP4* both showed copy number gains (Fig. 3a). *CCNE1* is involved in the cell cycle pathway (Fig. 6), and its amplification has been identified in multiple cancers. Among the known driver CNVs found in GB-NECs, the copy number of *MYC* increased the most (copy number 69.21). *MYC* is also frequently amplified in a variety of tumors. As described by Stine et al.,^34^ *MYC* is an enigmatic oncogene, in that it seems to affect all cellular processes. These genes with CNVs in GB-NECs may be potential therapeutic targets.

We discovered 23 genes with potential drug responses in GB-NECs. However, according to the annotation results, we found that most of these reported drug effects were related to mutations in the corresponding genes in a wide range of mutation sites, such as “oncogenic mutations”, rather than specific mutation sites. Moreover, these drug effects were reported in other tumors, not in GB-NEC. Therefore, our results have potential guiding roles in the clinical treatment of GB-NEC; however, more direct evidence is needed to confirm the effect of specific drugs. For example, according to previous studies, *ALK* mutations exert various effects on tumor development and the response to ALK TKI targeted therapy. Many oncogenic mutations in *ALK* have been identified.^35^ For example, Wang et al.^36^ found that the overexpression of H694R- or E1384K-mutant ALK leads to the hyperphosphorylation of ALK and the activation of downstream oncogenic signaling. Treatment with ALK inhibitors resulted in significantly improved survival in ALK-mutant-bearing mice.^36^ In contrast, various mutations, such as L1196M, G1269A, I1151T‐ins, G1202R, S1206Y, and I1171T, have been reported to be associated with resistance to specific ALK TKIs, which can be mediated by the activation of bypass pathways, including the ERBB pathway.^37,38^ It is still difficult to predict the oncogenic functions and response to different ALK inhibitors of different *ALK* mutations precisely by bioinformatic tools, although this is of great significance for therapeutic implications. However, as almost all the TKI resistance-related *ALK* mutations are located in the ALK kinase domain, we believe that the three *ALK* mutations identified in GB-NECs in our study are not resistant to TKIs. However, because the sensitivity to TKIs of the three *ALK* mutations identified in GB-NECs has not been reported previously, further experimental studies need to be conducted to validate their potential oncogenic roles and association with the response to ALK TKIs.

In GB-NECs, we found that *TGFB3* was mutated only in the N0 group; in contrast, *FAT4*, *PTPRT*, *ZNF728*, *ZNF845*, and *ZNF90* were mutated specifically in the N1/N2 group. As reviewed by Laverty et al.^39^ *TGFB3* may play a protective role against tumorigenesis in a range of tissues, including the skin, breast, oral, and gastric mucosa. *TGFB3* has a suppressor effect in the early stages of tumorigenesis according to preclinical data.^39^ Our results indicate an association between *TGFB3* mutations and early stage GB-NECs. *FAT4* is frequently mutated and known as a tumor suppressor in multiple tumors. FAT4 suppresses tumor growth by activating Hippo signaling.^40^ *PTPRT*, originally discovered as a primarily neurological protein, is frequently mutated in human cancers, including colon, lung, and gastric cancers. *PTPRT* plays an integral role in cell adhesion and intracellular signaling and was proven to be a tumor suppressor.^41,42^ Our data suggest that *FAT4* and *PTPRT* may have tumor-suppressive roles in GB-NECs. The roles of *ZNF728*, *ZNF845*, and *ZNF90* in tumors have not yet been reported and need to be further investigated in GB-NECs in future studies.

Mutations in *CTNNB1* were related to prolonged OS in GB-NEC patients. Two of the *CTNNB1* mutated GB-NEC samples (with *CTNNB1* p.Ser33Cys and p.Ser37Phe) showed the nuclear expression of β-catenin. β-Catenin is involved mainly in cell-to-cell adhesion and is a component of the Wnt signaling pathway. The deregulation of β-catenin signaling is crucial in the genesis of multiple tumors,^43^ and elevated levels of β-catenin activity are associated with *CTNNB1* mutations.^44^ S33 and S37 are hotspot mutation sites at exon 3 of *CTNNB1* and are the phosphorylation sites for GSK-3β. Mutations in exon 3 of *CTNNB1* can induce the accumulation of nuclear β-catenin and activate the canonical Wnt pathway.^44^ It is not very clear why the four patients with *CTNNB1* mutations had relatively good prognoses, which may need to be further investigated and validated in future studies. However, mutations in *CTNNB1* exon 3 may be potential markers for drugs targeting the Wnt pathway in GB-NECs.

We predicted six known somatic mutation signatures in GB-NECs: signatures 1, 3, 30, 6, 7, and 13. In the COSMIC database (https://cancer.sanger.ac.uk/cosmic/signatures_v2), signature 1 is characterized by an endogenous mutational process initiated by the spontaneous deamination of 5-methylcytosine. Signature 3 is associated with the failure of DNA double-strand break repair by homologous recombination. Signature 6 is associated with defective DNA mismatch repair and is found in tumors with microsatellite instability. Signature 7 is likely due to ultraviolet light exposure. Signature 13 is related to the activity of the AID/APOBEC family of cytidine deaminases. The etiology of signature 30 remains unknown. Signature 1 was also found in GBCs in our previous study,^12^ but signatures 3, 6, 7, 13, and 30 were discovered in GB-NECs in the current study. These signature-related biological processes may play a role in gene mutations and the occurrence of GB-NEC.

In summary, we have revealed mutations and potential somatic SNVs in GB-NEC. The large scale of genetic characteristics in GB-NEC may offer mechanistic insights for cancer diagnosis and therapeutic targets.

## Materials and methods

### Patient tissue collection, processing, and WES

In total, FFPE tumor samples from individual GB-NEC patients were collected from four hospitals: the Second Affiliated Hospital of Zhejiang University School of Medicine, Ganzhou People’s Hospital, Ningbo Ninth Hospital, and Ningbo Medical Center Lihuili Eastern Hospital. The FFPE tumor samples of GBC patients were collected from Xinhua Hospital Affiliated to Shanghai Jiao Tong University School of Medicine. This study was approved by the ethics committees of the participating hospitals. Clinical data associated with the samples were collected. The clinical information on GB-NEC and GBC patients is briefly summarized in Table 1 and Supplementary Table S1, respectively. Clinical staging was performed based on the eighth staging system of the American Joint Committee on Cancer. The FFPE tumor samples were pathologically verified. Subsequently, DNA was extracted to prepare the next-generation sequencing library.

**Table 1:** Clinical information of the GB-NEC patients

Sample_ID	Gender	Age	Family history	Tumor site	Jaundice	T stage	N stage	M stage	TNM stage	Differentiation	Overall survival status	Months to last follow up
GBNEC_1	Male	66	No	Bottom	Yes	3	1	0	IIIB	G3	Dead	11.03
GBNEC_2	Female	50	No	Bottom	No	4	1	0	IVB	G3	Dead	4.10
GBNEC_3	Female	65	No	Body	No	2a	0	0	IIA	G2	Dead	4.67
GBNEC_4	Male	54	No	Body	No	3	0	1	IVB	G2	Alive	124.73
GBNEC_5	Female	82	No	Bottom	No	3	2	0	IVB	G3	Dead	6.33
GBNEC_6	Female	65	No	Neck	No	1b	0	0	I	G2	Alive	44.23
GBNEC_7	Female	72	No	Neck	Yes	3	2	0	IVB	G3	Dead	2.87
GBNEC_8	Female	68	No	Neck	Yes	4	1	1	IVB	G3	Dead	1.70
GBNEC_9	Female	54	No	Neck and body	No	2a	0	0	IIA	G3	Alive	32.27
GBNEC_10	Male	66	No	Neck	No	1b	0	0	I	G3	Alive	35.30
GBNEC_11	Female	68	No	Neck	No	2a	2	0	IVB	G3	Dead	2.83
GBNEC_12	Male	59	No	Neck	Yes	3	1	0	IIIB	G3	Dead	20.07
GBNEC_13	Female	64	No	Neck and body	No	3	0	0	IIIA	G3	Dead	13.67
GBNEC_14	Female	60	No	Bottom	No	3	2	0	IVB	G3	Dead	22.67
GBNEC_15	Female	59	No	Bottom	No	3	2	0	IVB	G3	Alive	6.83

DNA length and integrity were confirmed on an Agilent 2100 Bioanalyzer (Agilent, Santa Clara, USA). The library was constructed as described in our previous study and according to the procedure recommended by Illumina.^12^ Briefly, genomic DNA was fragmented, purified, end-repaired, adenylated on the 3′ ends, ligated with indexed pair-end adaptors, purified again, and amplified by polymerase chain reaction (PCR). Exome capture was performed using Agilent SureSelect Human All Exon V6 Probes (Agilent, Santa Clara, USA). After PCR amplification, purification, library validation, normalization, and pooling, the libraries were sequenced with the Illumina HiSeq Series Analyzer, yielding 300 base pairs (2 × 150) from the final library fragments.

### WES data analysis

Adapter trimming, BWA read mapping and GATK processing was conducted as described in our previous study.^12,45–47^ The resulting bam files from BWA have also been uploaded to the Sequence Read Archive (SRA) database (https://www.ncbi.nlm.nih.gov/sra). We uploaded the WES data of GBC and GB-NEC as separate projects. The accession number of the GB-NEC project in this database is PRJNA636203, and the accession number of the GBC project is PRJNA638698. VarScan 2 software^48^ was applied for variant detection with default parameters, except for “--min-coverage 15 --min-var-freq 0.08”. The fpfilter module of VarScan 2 was used to remove false-positive variations. We also used our inner database of false-positive mutations to conduct an additional filter. VEP, SnpEff, and GEMINI software^49–51^ were applied to annotate the variants using information from publicly available databases, including ClinVar and ExAc. After annotation, we further filtered the mutations using the following criteria: (1) the impact of the mutations predicted by SnpEff was “HIGH” or “MODERATE”; (2) the depth of the alternative allele was >5; and (3) the frequency of the alternative allele in the population (max_aaf_all provided by GEMINI) was <0.0005 and such mutations were defined as “rare mutations” in the general population.

### Summary of WES data analysis results

The genome-wide distribution of the mutations was visualized as a CIRCOS figure using ClicO FS.^52^ Maftools^53^ was applied for the analysis, statistics, and visualization of mutations from MAF files, including the variant classifications, proportions of SNVs, oncoplots of genes with the most frequent mutations, lollipop plots of genes, mutually exclusive or cooccurring set of genes, Pfam domains with significant mutations, mutation comparisons of the two cohorts, and known oncogenic signaling pathways in the TCGA cohorts. Heatmap and Venn diagram figures were made by TBtools.^54^

### Pathway enrichment analysis

Pathway enrichment analysis was performed by using the ConsensusPathDB (http://cpdb.molgen.mpg.de).^55^ The overrepresentation analysis module of the web-based software was applied. The KEGG database was used as the pathway reference database. The analysis criteria were as follows: minimum overlap with input list, 2; and *p* value cutoff 0.01 for the genes with mutations and 0.05 for the genes with CNVs.

### SMG analysis

We used MutSigCV^56^ to detect SMGs in the 15 GB-NEC samples. MutSigCV software was run with default covariate tables to calculate gene mutational significance. Silent mutations (predicted by SnpEff as “LOW”) with a < 0.0005 frequency of the alternative allele in the population were also included in the analysis. Genes with a *p* value ≤ 0.05 in the output file were involved in the SMGs and used for subsequent analysis. In addition, genes for which information on expression levels and HiC-based chromatin state estimation was unavailable in the MutSigCV database were removed from the results.

### CNV analysis

We used our own pipeline to analyze CNVs. Briefly, in each sample and for each gene, we calculated the number of sequencing reads of all exons by extracting the information from the bam files according to the genomic locations of exons downloaded from the UCSC Genome Browser (https://genome.ucsc.edu). Subsequently, the data were balanced by the average sequencing depth of the corresponding sample. The resulting data were then compared with our internal control dataset generated from the sequencing data of peripheral blood leukocytes, and the relative copy numbers of the exons of each gene were calculated. Then, to avoid false positives, we focused only on genes with more than two exons. Genes were determined as having copy number gain if all of the exons had a copy number >2.8. Correspondingly, genes were determined as having copy number loss if all of the exons had a copy number <1.2. The distributions of genes with CNVs among samples were summarized with Oncoprinter.^57^

### Somatic mutation prediction

We first used ISOWN (Identification of SOmatic mutations Without matching Normal tissues),^58^ a supervised machine learning algorithm, to predict potential somatic SNVs. Briefly, the original SNVs generated from the process described above were annotated with COSMIC (v69), dbSNP (v142), ExAC (release 2), PolyPhen WHESS (released in 2015), and Mutation Assessor (released in 2013). The somatic SNVs were then predicted by ISOWN using default criteria. Subsequently, we applied another single-sample variant caller, SomVarIUS,^59^ to further analyze the somatic mutations. Somatic SNVs predicted by both ISOWN and SomVarIUS were subjected to the subsequent analysis.

### Mutation signature analysis

We used Mutalisk^60^ to analyze the mutation signatures of the somatic SNVs. A linear regression test was applied as the maximum likelihood estimation method for the decomposition of mutation signatures referring to the 30 COSMIC signatures. The best decomposition model was ranked and chosen based on the BIC.

### Statistical analysis

Survival analysis was performed using GraphPad Prism version 8.2.1 for Mac (GraphPad Software, La Jolla California USA, www.graphpad.com). The comparison of survival curves was performed using the log-rank test. The *p* value and hazard ratio of the log-rank test were also calculated. The comparison of RB1/NAB2 expression between RB1/NAB2 wild-type and mutant samples was also performed by GraphPad Prism using an unpaired *t* test.

### Immunohistochemistry

Immunohistochemical staining was performed using a standard immunoperoxidase staining procedure. Antibodies against RB1 and NAB2 were used at dilutions of 1:150 and 1:200, respectively. Immunoreactivity was semiquantitatively scored on a scale from 0 to 4+ as follows: 0 (<10%), 1+ (10–25%), 2+ (>25–50%), 3+ (>50–75%), or 4+ (>75%).^61^ Strong and intact nuclear expression of RB1 in endothelial cells served as an internal positive control. Moderate nuclear and cytoplasmic expression of NAB2 in glandular epithelial cells also served as a positive control. Antibodies against β-catenin were used at a dilution of 1:100. The nuclear expression of β-catenin was compared between the *CTNNB1* wild-type and mutant samples.

## Supplementary information

Supplementary Materials

Supplementary table-S1

Supplementary table-S2

Supplementary table-S3

Supplementary table-S4

Supplementary table-S5

Supplementary table-S6

Supplementary table-S7

Supplementary table-S8

Supplementary table-S9

Supplementary table-S10

Supplementary table-S11

Supplementary table-S12

Supplementary table-S13

Supplementary table-S14

Supplementary table-S15

Supplementary table-S16

Supplementary table-S17

Supplementary table-S18

Supplementary table-S19

## Data Availability

The original bam files have been uploaded to the Sequence Read Archive (SRA) database (https://www.ncbi.nlm.nih.gov/sra). The accession number of the GB-NEC project in this database is PRJNA636203, and the accession number of the GBC project is PRJNA638698. Other data supporting the findings of this study are available within the paper and its Supplementary materials.
