# Supplementary material for: Whole-exome mutational landscape of neuroendocrine carcinomas of the gallbladder
Source: Signal Transduct Target Ther. 2021 Feb 10;6:55. doi: 10.1038/s41392-020-00412-3 (PMC7873252; doi:10.1038/s41392-020-00412-3)
Supplement: Supplementary file 1 — Supplementary Materials [file 41392_2020_412_MOESM1_ESM.pdf]

Supplementary Materials for

Whole-Exome Mutational Landscape of Neuroendocrine Carcinomas of the Gallbladder

Fatao Liu, Yongsheng Li, Dongjian Ying, Shimei Qiu, Yong He, Maolan Li, Yun Liu, Yijian Zhang, Qin zhu, Yunping Hu, Liguo Liu, Guoqiang Li, Weihua Pan, Wei Jin, Jiasheng Mu, Yang Cao, Yingbin Liu

Correspondence to: mujiasheng@xinhuamed.com.cn; 2316029@zju.edu.cn; laoniulyb@shsmu.edu.cn

This PDF file includes:

Supplementary Figures. S1 to S20

Other Supplementary Materials for this manuscript include the following:

Data S1 to S19 (separate file)

Supplementary Table S1 Clinical information on the 22 GBC patients.
Supplementary Table S2 Detailed information on the mutations detected in 15 GB-NECs.
Supplementary Table S3 Detailed information on known hotspot mutations detected in GB-NEC and GBC samples.
Supplementary Table S4 SMG analysis results in GB-NECs.
Supplementary Table S5 List of SMGs in the four reported kinds of neuroendocrine cancers.
Supplementary Table S6 Detailed information on the mutations detected in 22 GBCs.
Supplementary Table S7 Pathway analysis results of genes mutated in GB-NECs but not GBCs.
Supplementary Table S8 Pathway analysis results of genes mutated in both GB-NECs and GBCs.
Supplementary Table S9 CNV events detected in GB-NEC samples.
Supplementary Table S10 CNV events detected in GBC samples.
Supplementary Table S11 Pathway analysis results of genes with a copy number gain in GB-NECs.
Supplementary Table S12 Pathway analysis results of genes with a copy number gain in GBCs.
Supplementary Table S13 Pathway analysis results of genes with a copy number loss in GB-NECs.
Supplementary Table S14 Pathway analysis results of genes with a copy number loss in GBCs.
Supplementary Table S15 Detailed information on the potentially pathogenic mutations detected in 15 GB-NEC samples.
Supplementary Table S16 Detailed information on the potentially pathogenic mutations detected in 22 GBC samples.

Supplementary Table S17 Detailed information on mutations in genes with a potential drug response in GB-NECs.

Supplementary Table S18 Detailed information on mutations in genes with a potential drug response in GBCs.

Supplementary Table S19 Detailed information on potential somatic SNVs.

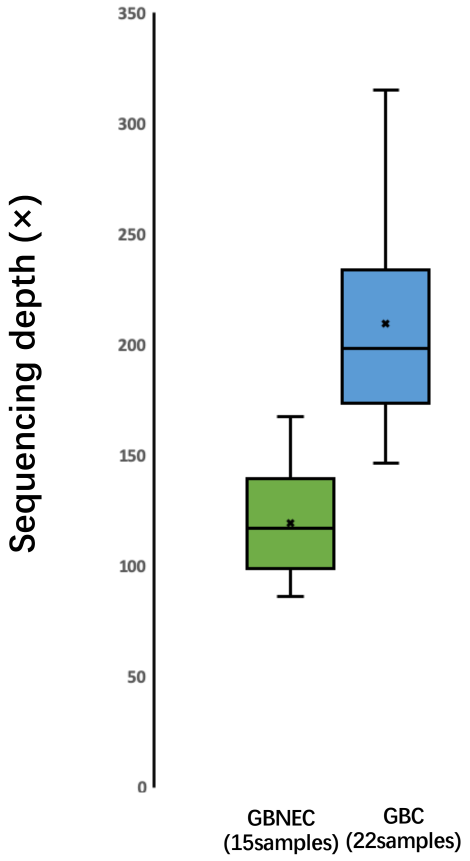

Figure. S1.

The sequencing depth of GB-NECs and GBCs.

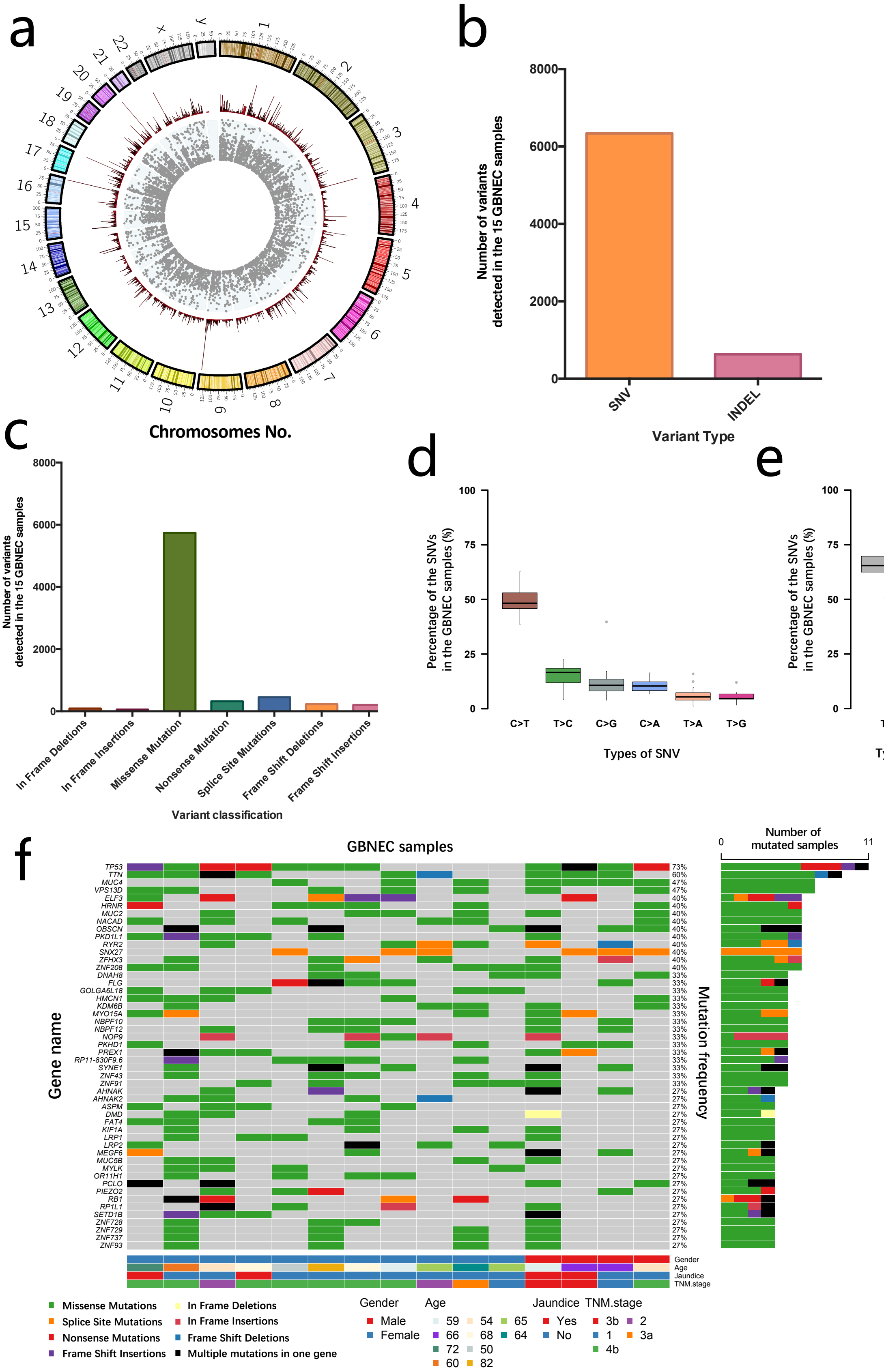

Figure. S2.

Characteristics of mutations and frequently mutated genes detected in 15 GB-NEC samples. (a) Genome-wide SNVs and INDELs discovered. From the outer circle to the inner circle: panel I: chromosome number and location; panel II: sequencing depth of the mutations; panel III: alternative allele frequency of the mutations. (b) Histograms of the number of SNVs and INDELs. (c) Histograms of the number of detailed mutation classifications. (d, e) Boxplot of the fraction of the types of SNVs, including 6 types of SNVs and transitions vs transversions. (f) Top 50 most frequently mutated genes.

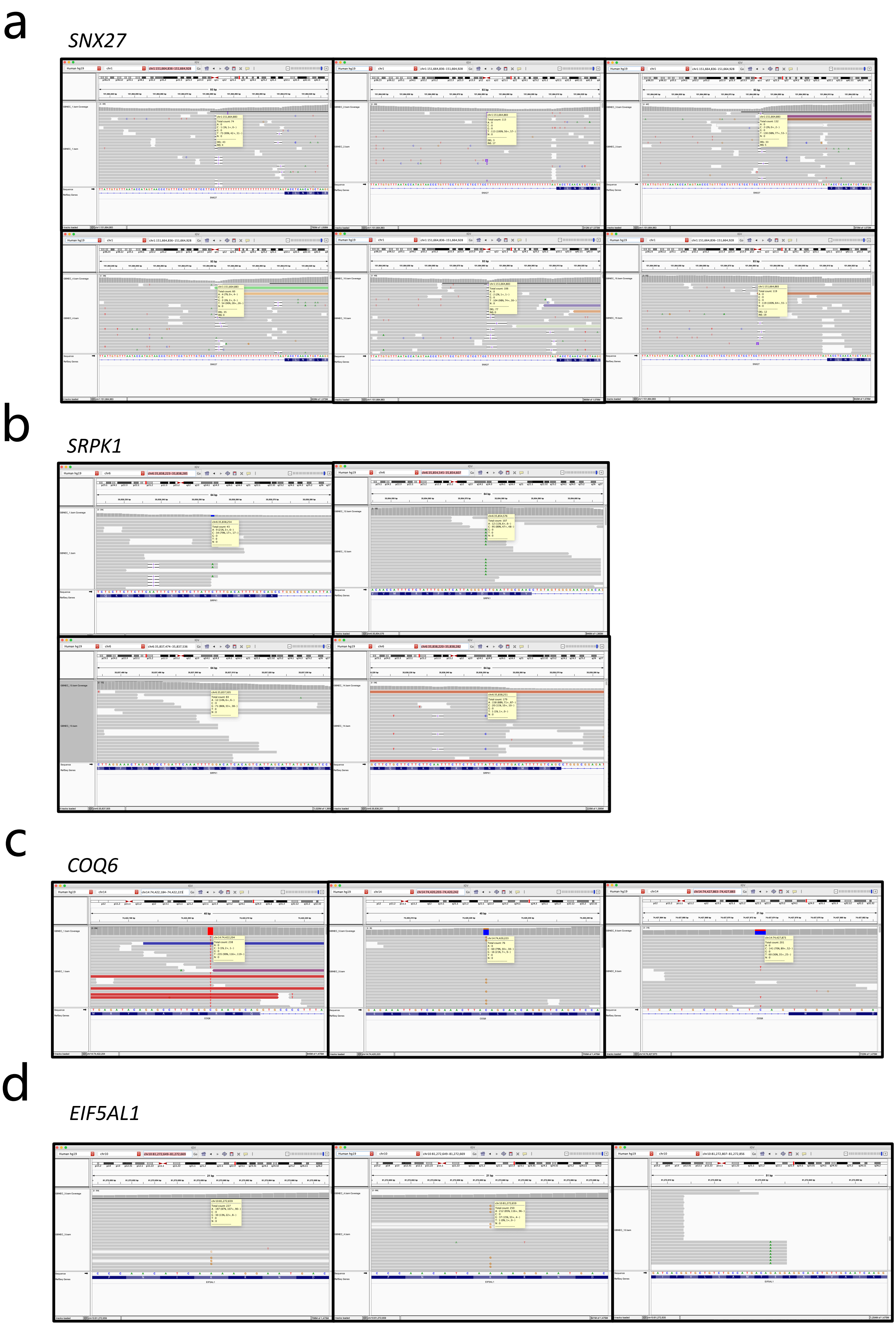

Figure. S3.

Detailed mutation information of several SMGs by IGV.

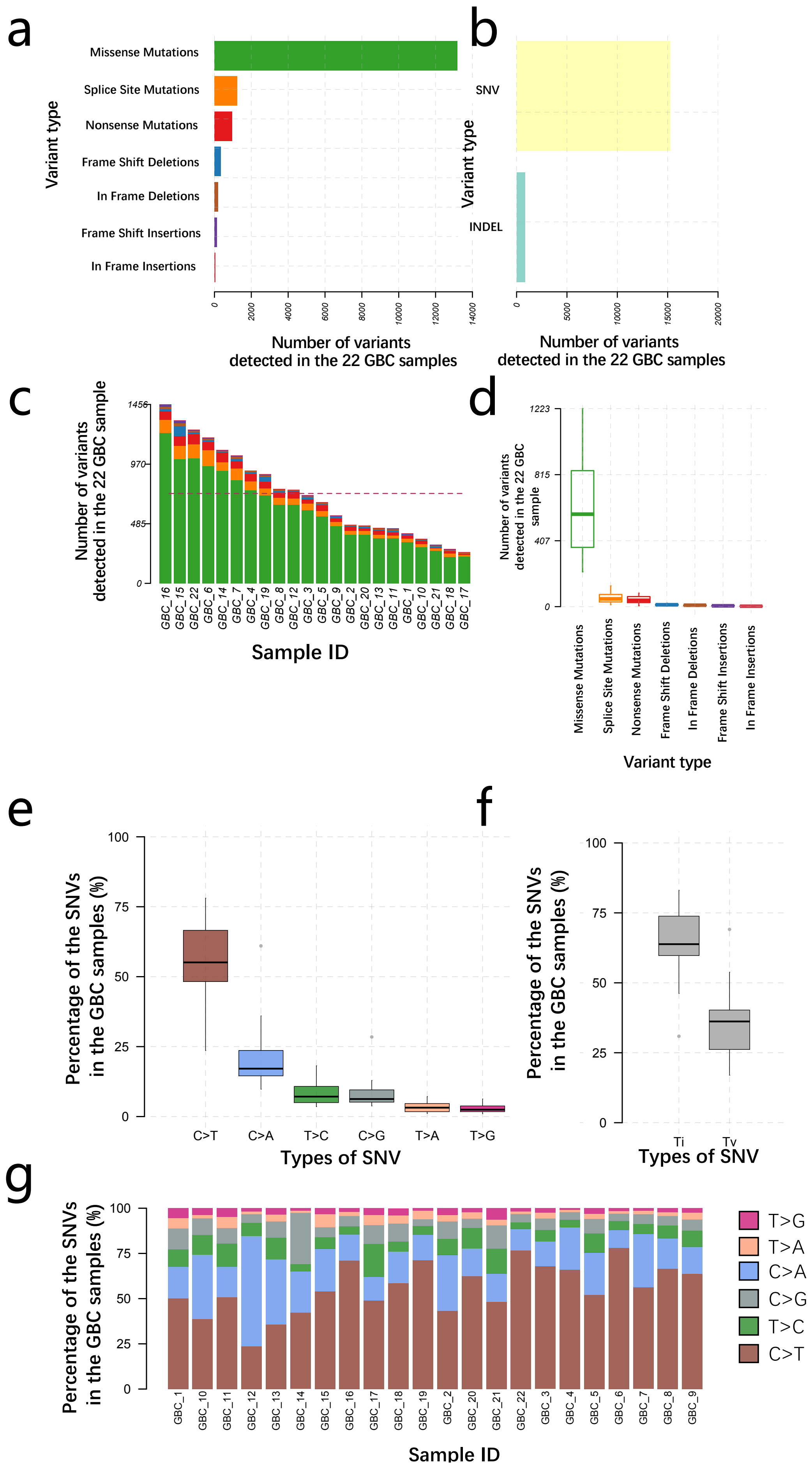

Figure. S4.

Basic characteristics of mutations detected in 22 GBC samples.

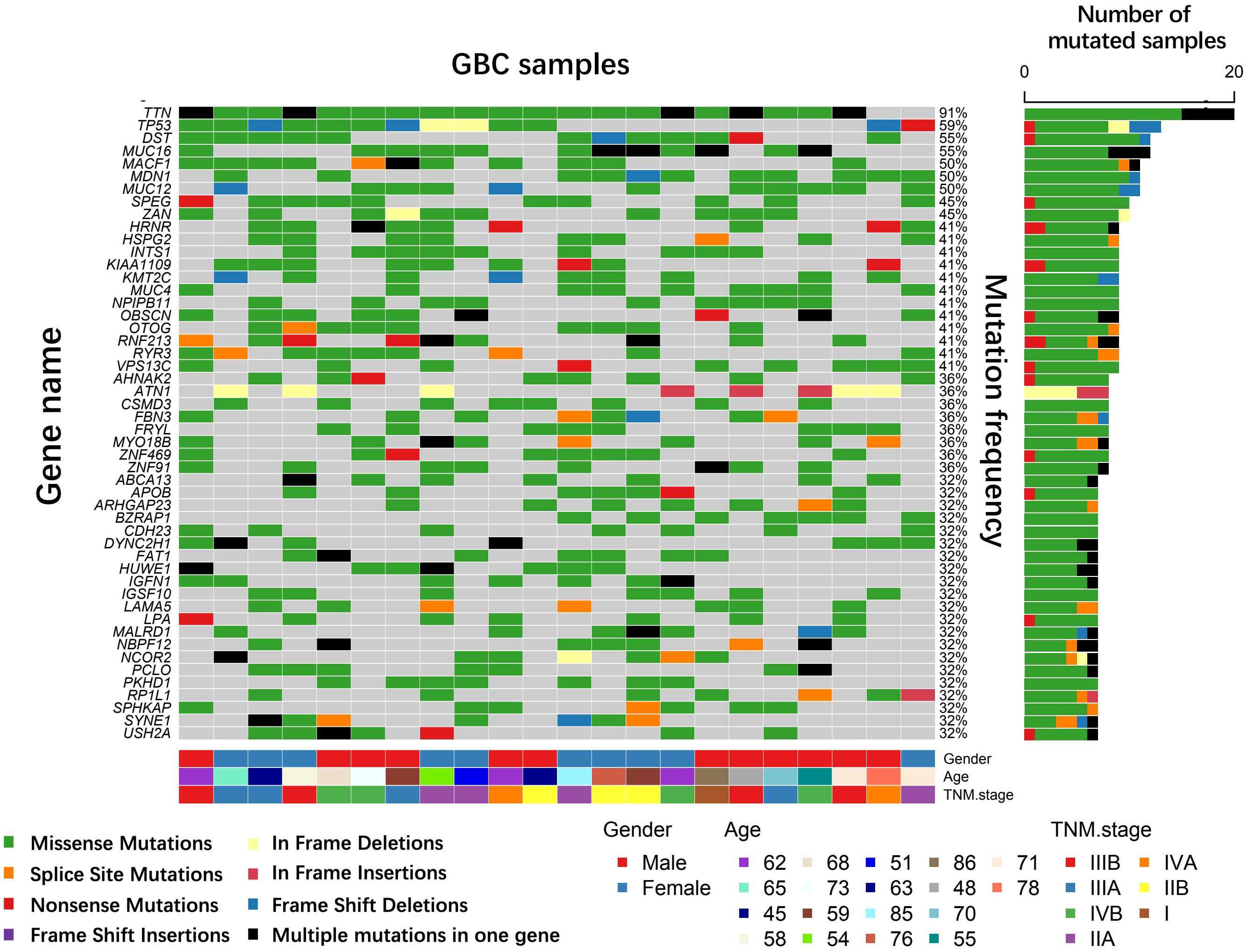

Figure. S5.

Detailed information on the mutations in the top 50 most frequently mutated genes in 22 GBC samples.

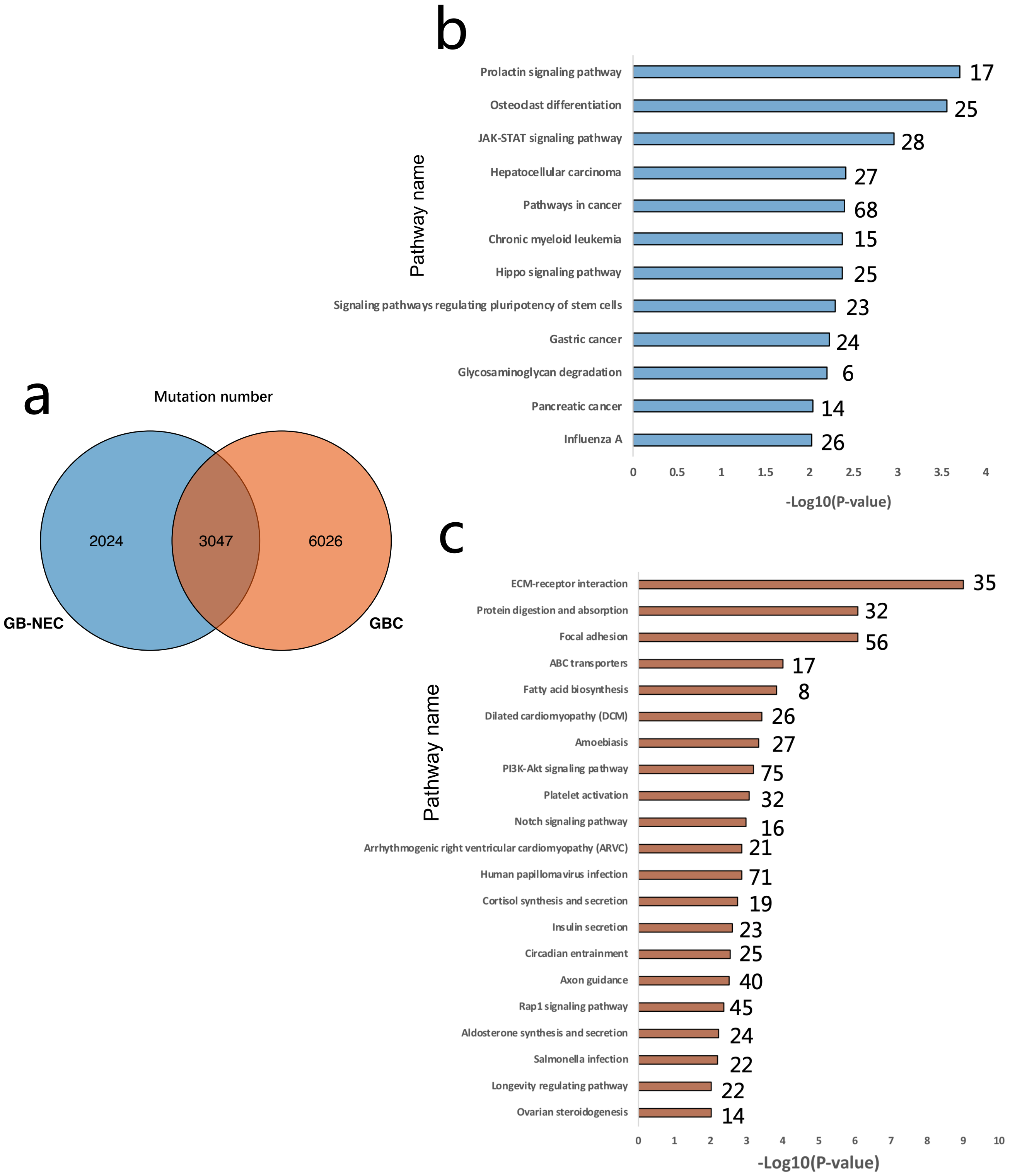

Figure. S6.

(a) Venn diagram of mutations detected in GB-NECs and GBCs. (b) Pathway enrichment analysis of genes mutated specifically in GB-NECs. The length of the column represents the -log10(p-value) of the enriched pathways. The number on the right side of the column represents the number of mutated genes detected in the pathway. (c) Pathway enrichment analysis of genes mutated in both GB-NECs and GBCs.

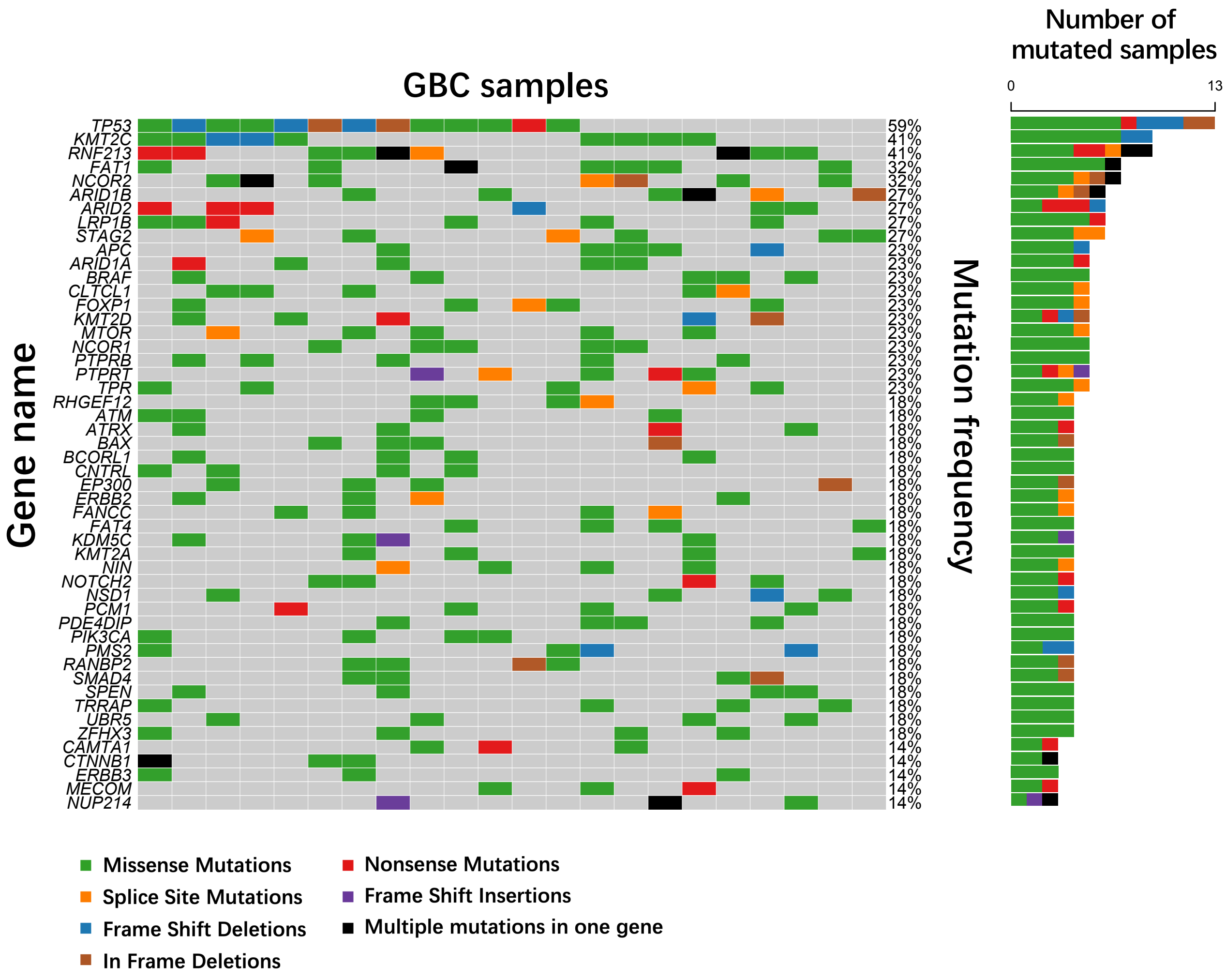

Figure. S7.

The mutation information of the top 50 cancer-related genes by mutation frequency in GBCs.

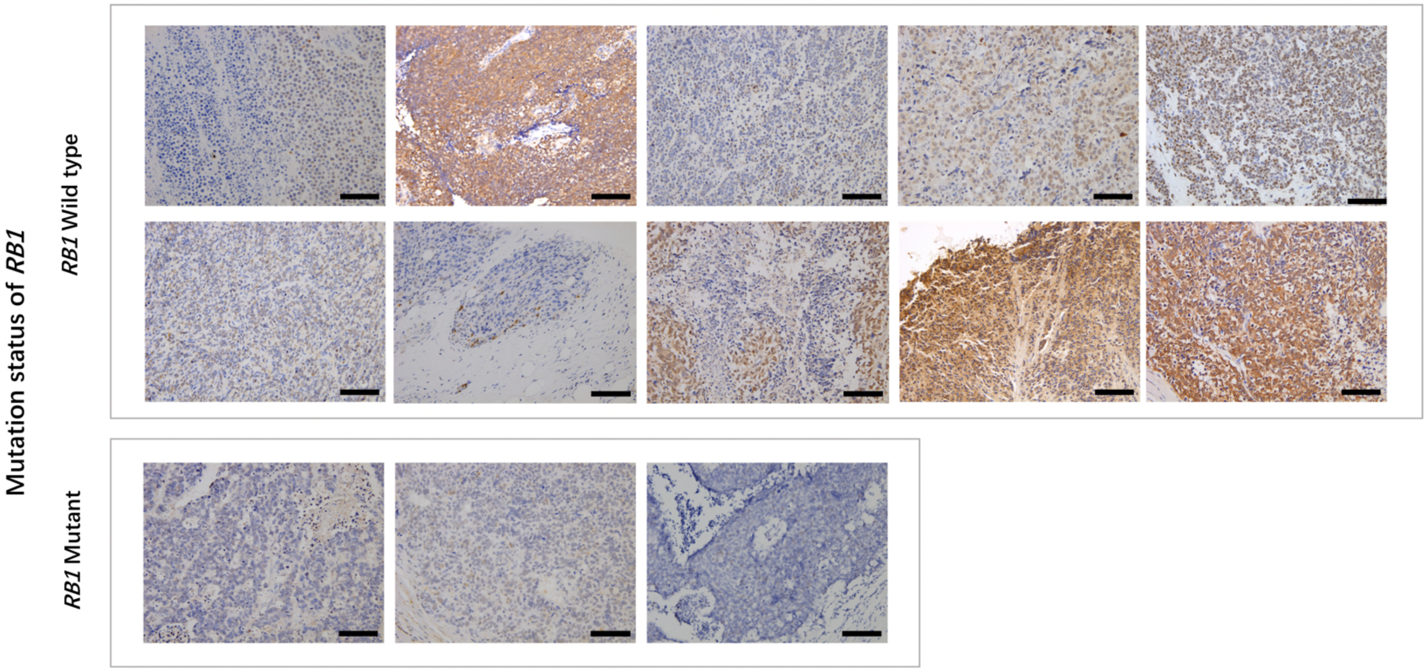

Figure. S8.

IHC results (magnification 200×) of RB1 in *RB1* wild-type/mutant GB-NEC samples. Scale bars in the figures indicate 100 μm.

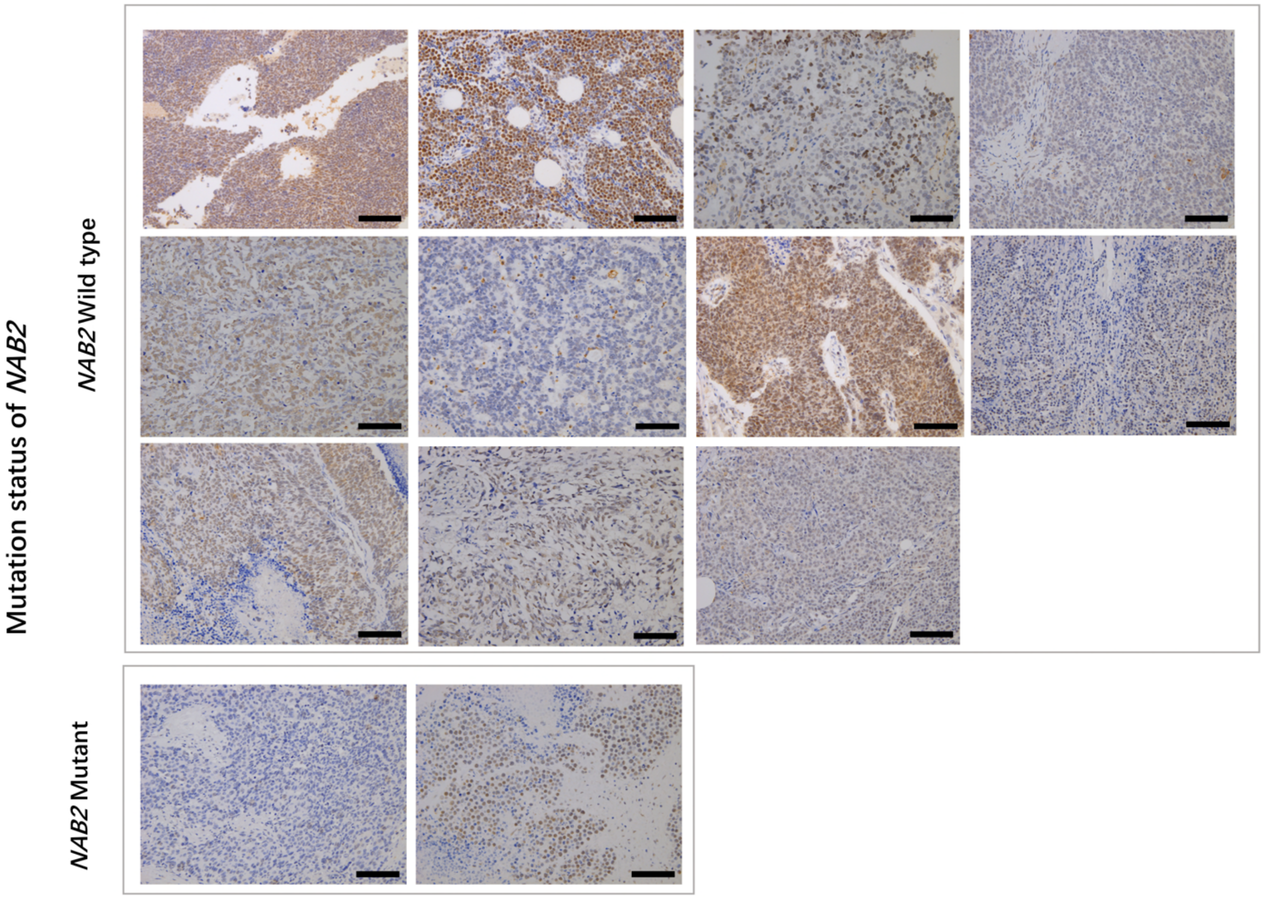

Figure. S9.

IHC results (magnification 200×) of NAB2 in *NAB2* wild-type/mutant GB-NEC samples. Scale bars in the figures indicate 100 μm.

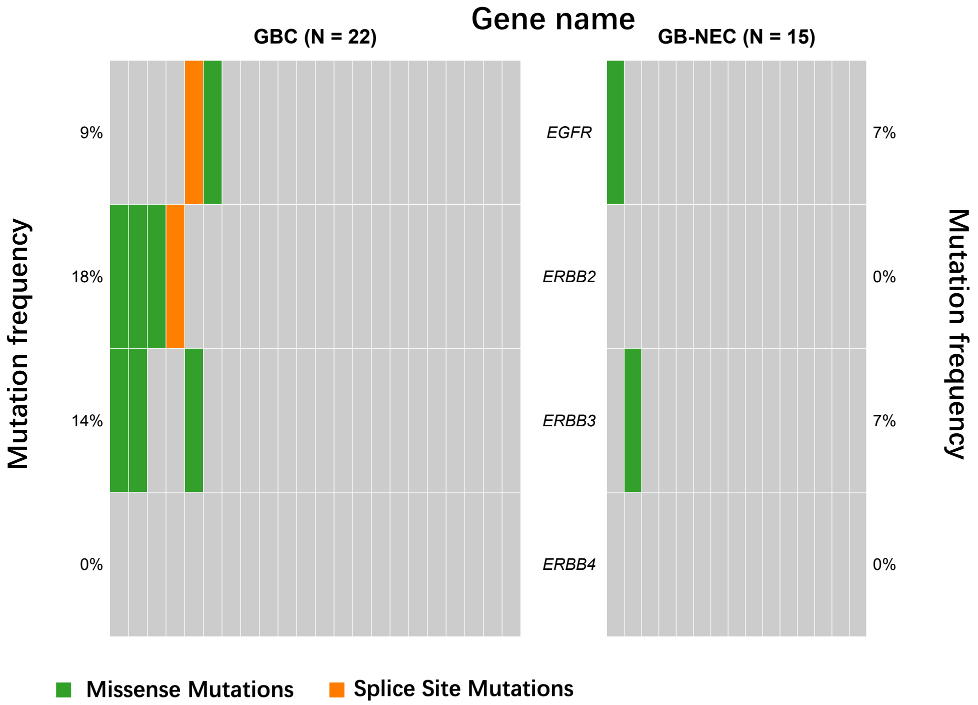

Figure. S10.

The mutation information of the *ERBB* family in GB-NECs and GBCs.

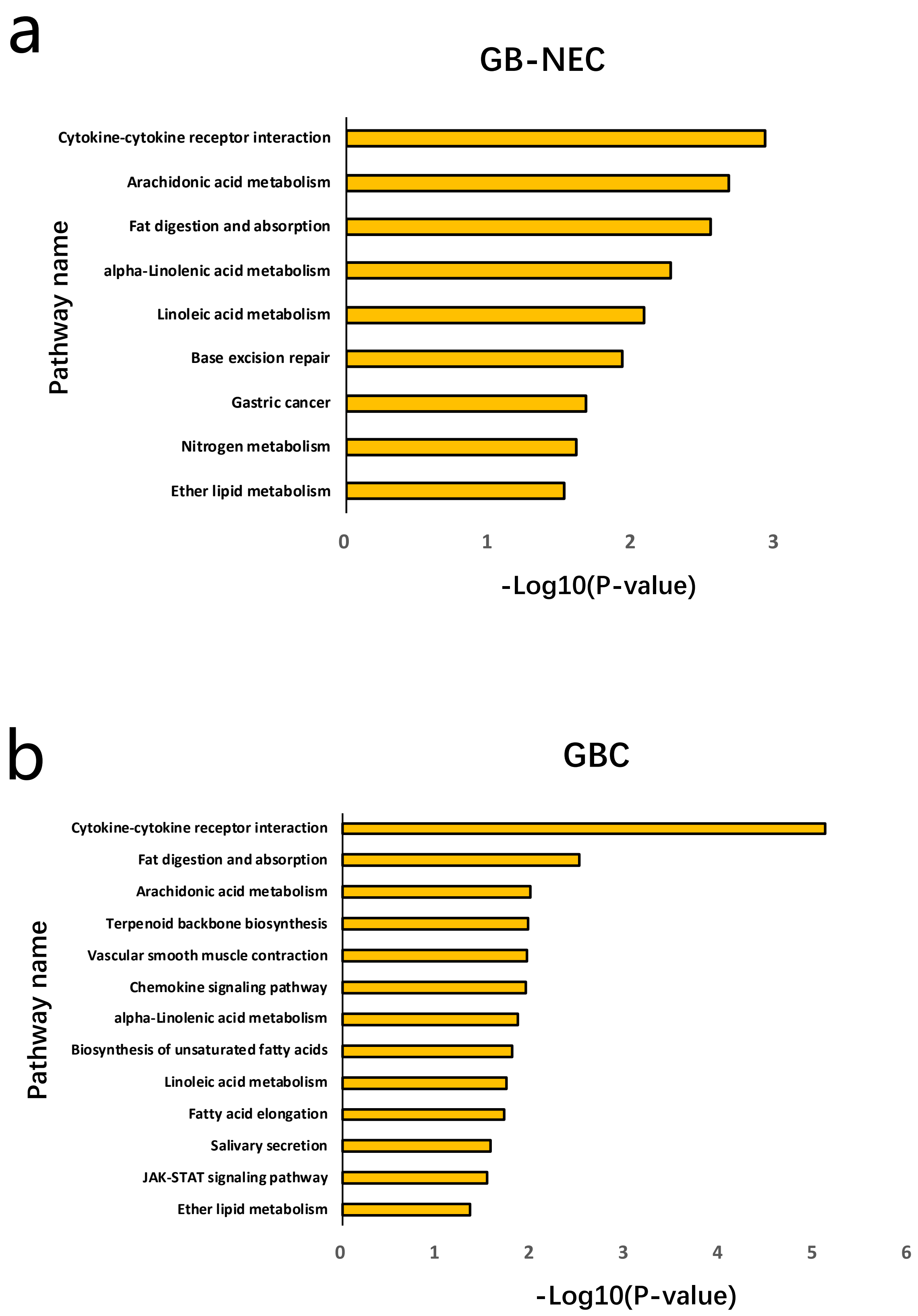

Figure. S11.

Combined enrichment analysis of genes with copy number gain and loss in GB-NECs and GBCs.

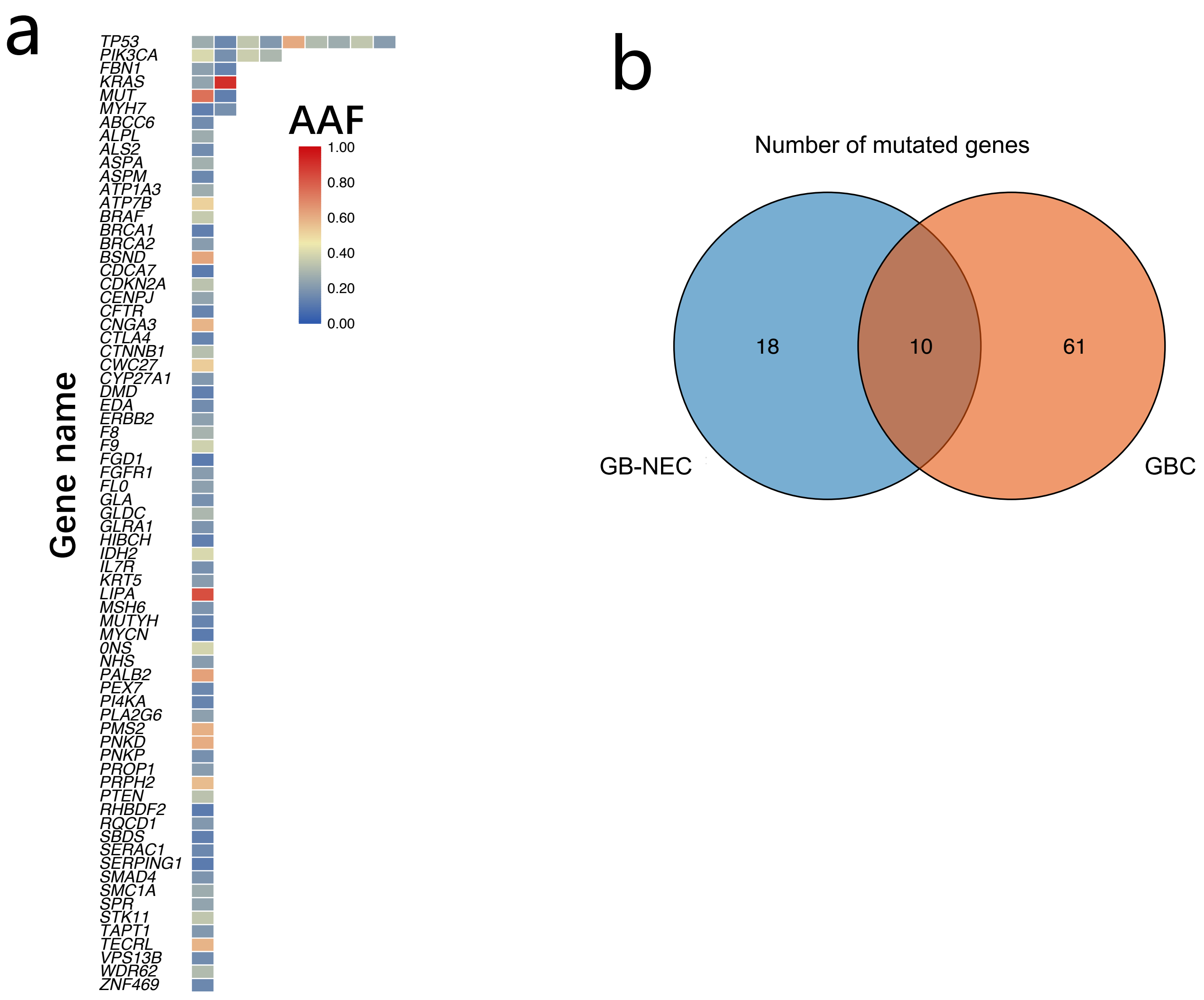

Figure. S12.

(a) Detailed information on the potential pathogenic mutations detected in 22 GBC samples. (b) Overlap of genes with disease-related mutations detected in GB-NECs and GBCs.

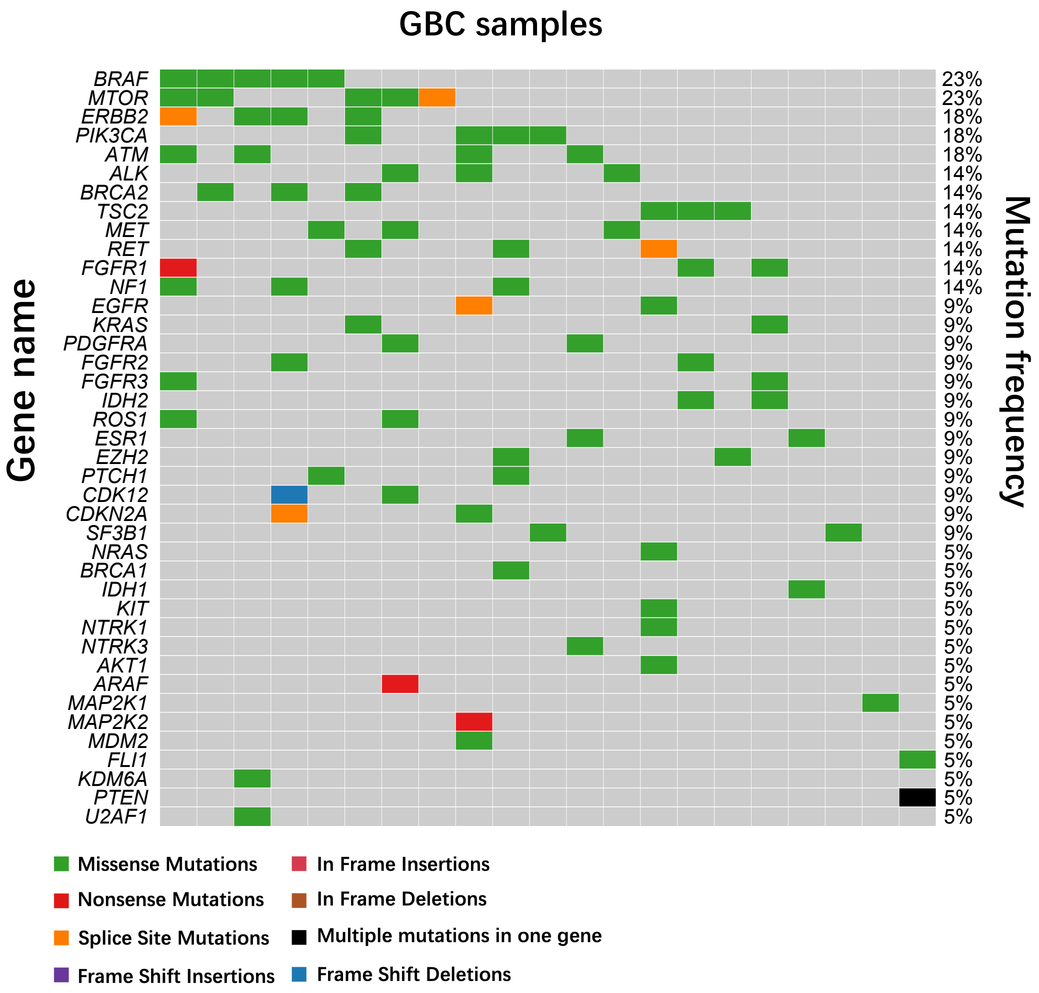

Figure. S13.

Detailed mutation information of genes mutated in the 22 GBC samples and with potential clinically actionable mutations recorded in the OncoKB database.

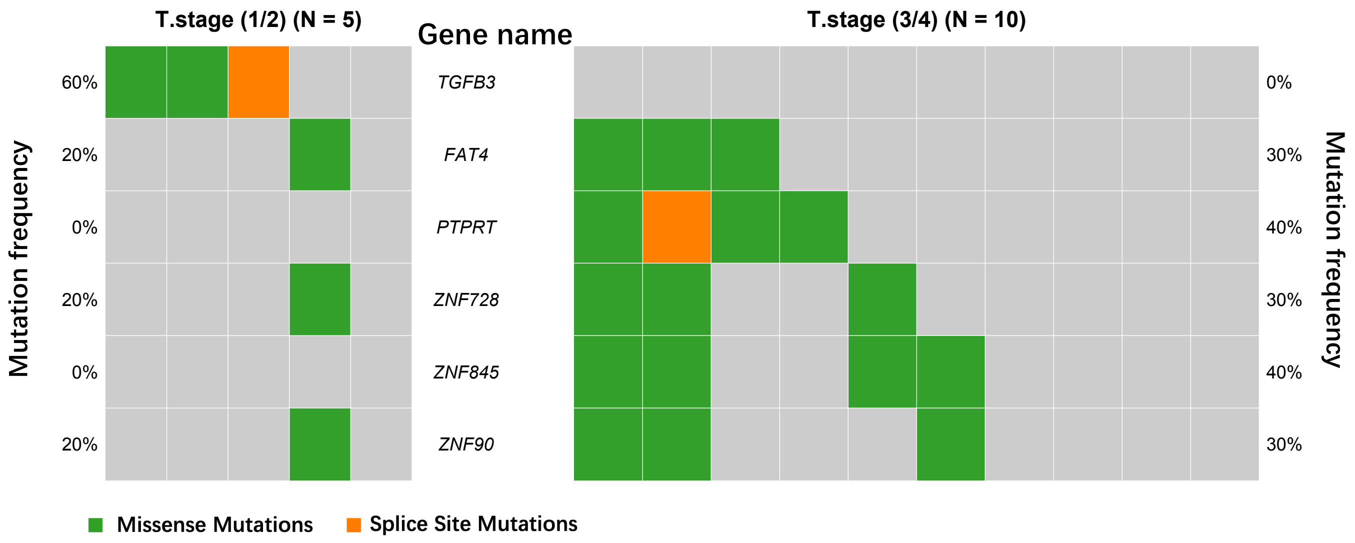

Figure. S14.

Mutations of N-stage related genes in GB-NECs of T1/T2 and T3/T4 stages.

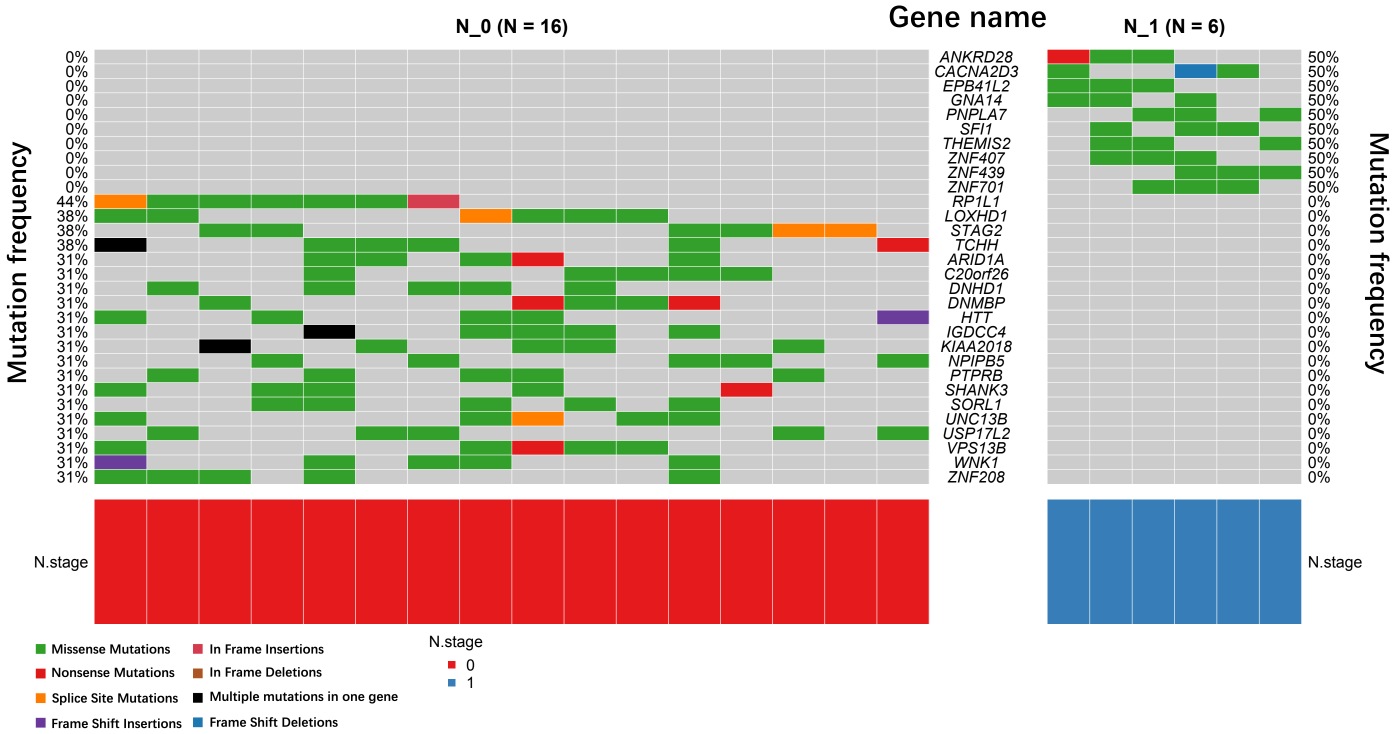

Figure. S15.

Genes specifically mutated in GBCs with the N0 and N1/N2 stages. Genes with mutation frequency > 30% in each group are shown.

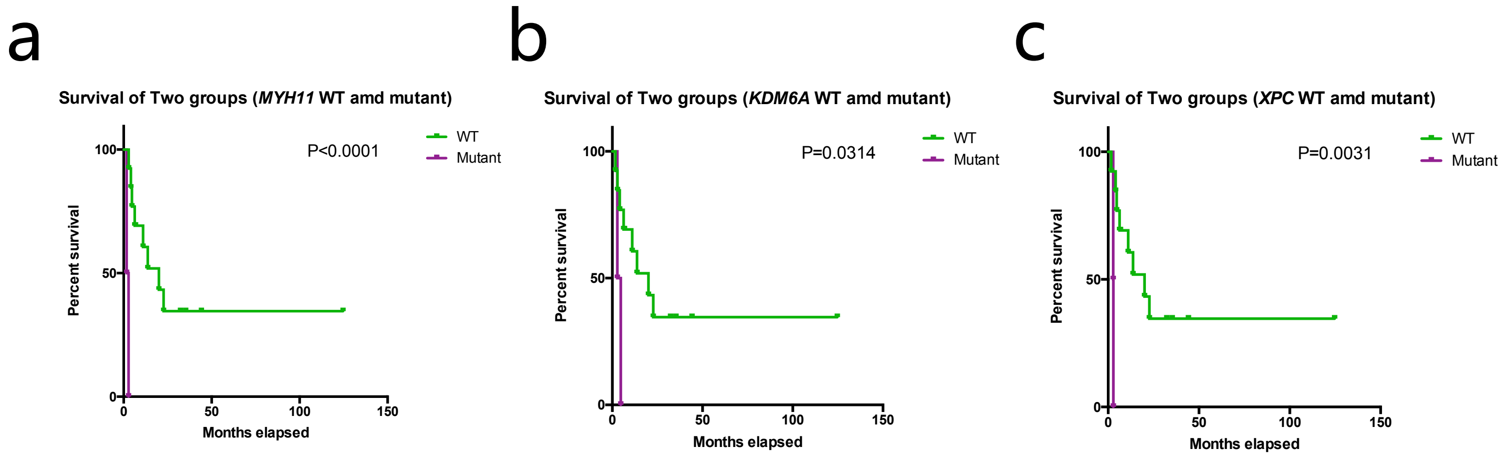

Figure. S16.

Survival analysis of patients with *MYH11/KDM6A/XPC* mutations respectively compared with those without mutations of these genes.

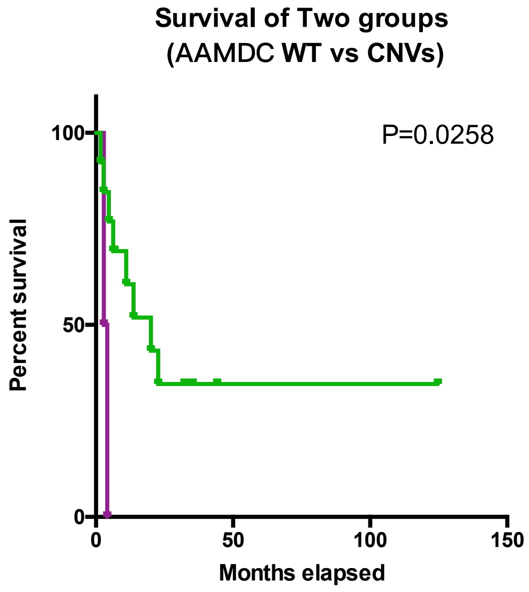

Figure. S17.

Survival analysis of GB-NEC patients with CNVs of *AAMDC* compared with those with wild type *AAMDC*.

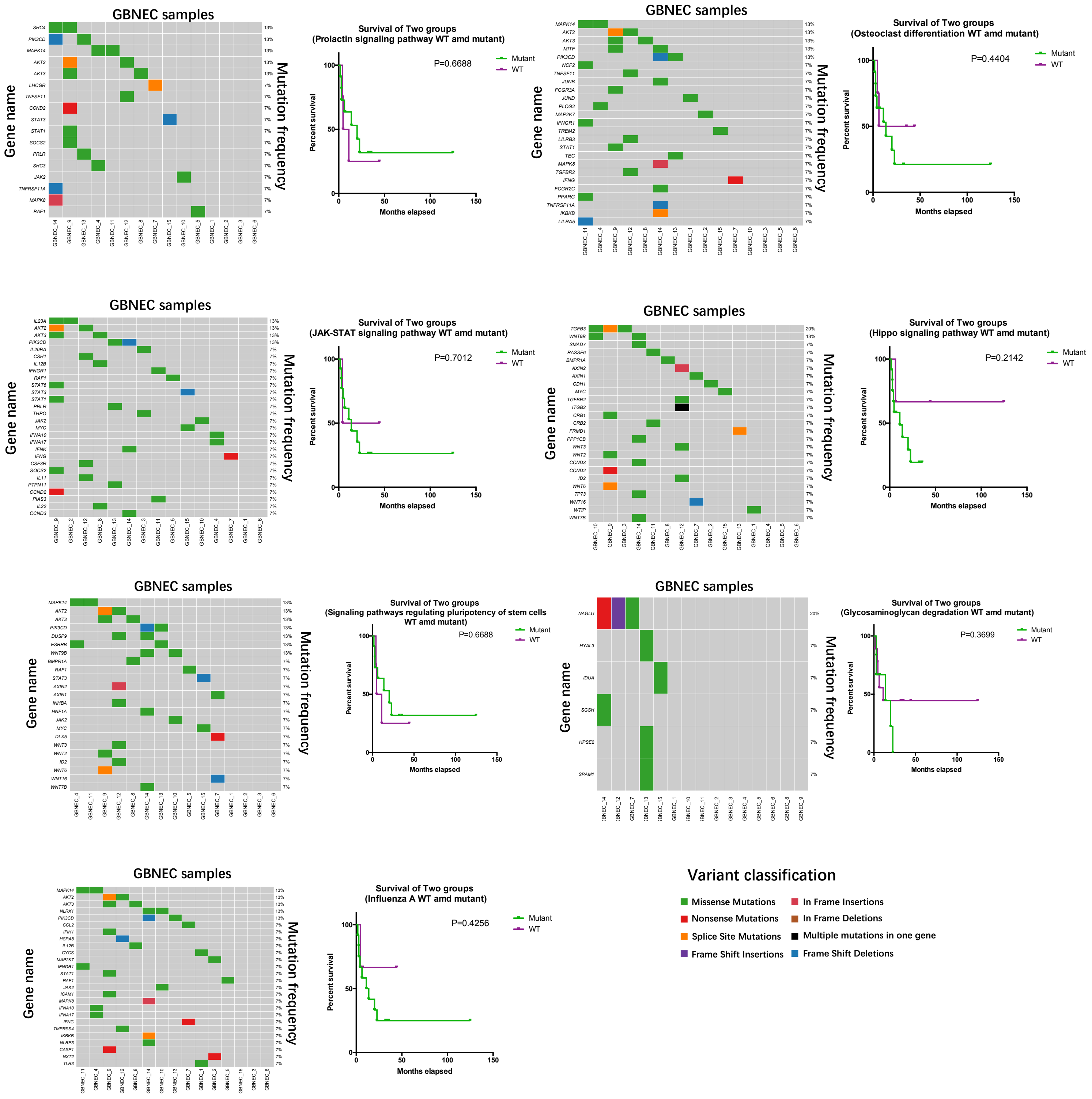

Figure. S18.

Mutation frequency and survival analysis of enriched signaling pathways of genes specifically mutated in GB-NECs.

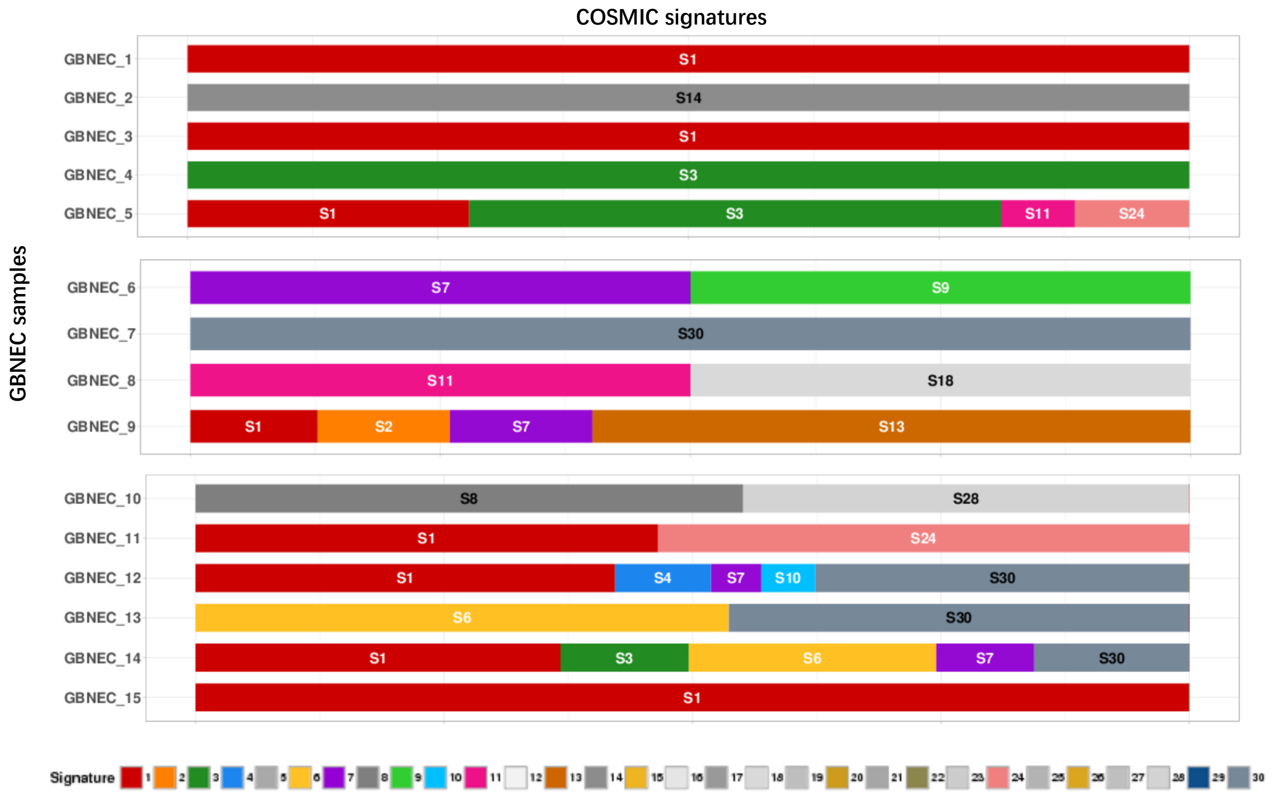

Figure. S19.

The mutation signatures discovered in each GB-NEC sample.

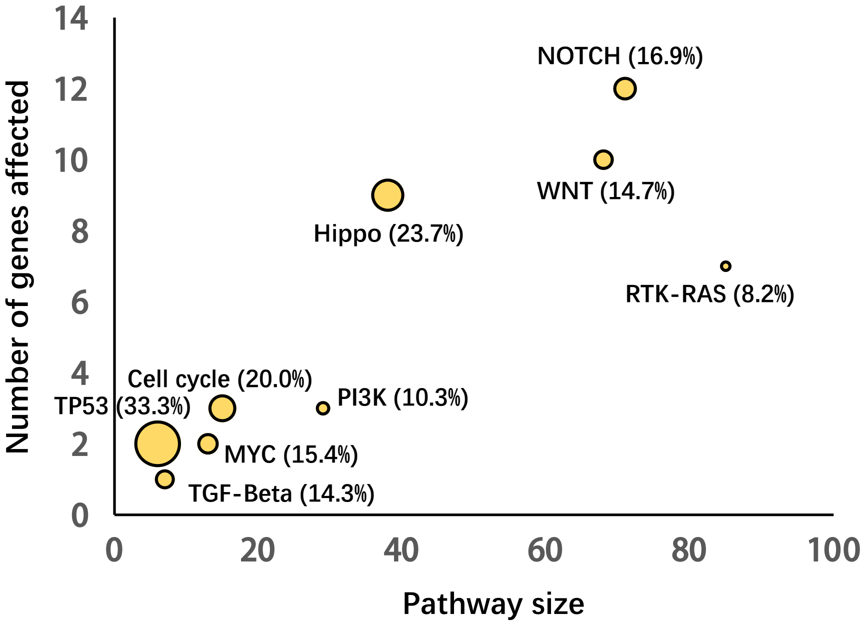

Figure. S20.

Enrichment analysis of known oncogenic signaling pathways in TCGA cohorts. The horizontal axis represents the total number of genes in the pathway and the vertical axis represents the number of genes mutated in the corresponding pathway. The size of the circle represents the percentage of mutated genes in all genes of the pathway, the detailed number of which is also labeled after the pathway name.
